# Evidence from 43 countries that disease leaves cultures unchanged in the short-term

**DOI:** 10.1038/s41598-023-33155-6

**Published:** 2024-03-18

**Authors:** Gian Luca Pasin, Aron Szekely, Kimmo Eriksson, Andrea Guido, Eugenia Polizzi di Sorrentino, Giulia Andrighetto

**Affiliations:** 1 Department of Social and Political Sciences, University of Milan, Milan, Italy; 2 Collegio Carlo Alberto, Turin, Italy; 3 Institute of Cognitive Sciences and Technologies, National Research Council of Italy, Rome, Italy; 4 Center for Cultural Evolution, Stockholm University, Stockholm, Sweden; 5 CEREN EA 7477, Burgundy School of Business, Université Bourgogne Franche-Comté, Dijon, France; 6 Institute for Futures Studies, Stockholm, Sweden; 7 Malardalens University, Västerås, Sweden

**Keywords:** Cultural evolution, Human behaviour

## Abstract

Did cultures change shortly after, and in response to, the COVID-19 outbreak? If so, then in what way? We study these questions for a set of macro-cultural dimensions: collectivism/individualism, duty/joy, traditionalism/autonomy, and pro-fertility/individual-choice norms. We also study specific perceptions and norms like perceived threats to society (e.g. immigration) and hygiene norms. We draw on Evolutionary Modernization Theory, Parasite Stress Theory, and the Behavioural Immune System, and existing evidence, to make an overarching prediction: the COVID-19 pandemic should increase collectivism, duty, traditionalism, conformity (i.e. pro-fertility), and outgroup prejudice. We derive specific hypotheses from this prediction and use survey data from 29,761 respondents, in 55 cities and 43 countries, collected before (April–December 2019) and recently after the emergence of COVID-19 (March–July 2020) to test them. We exploit variation in disease intensity across regions to test potential mechanisms behind any changes. The macro-cultural dimensions remained stable. In contrast, specific perceptions and norms related to the pandemic changed: norms of hygiene substantially increased as did perceived threats related to disease. Taken together, our findings imply that macro-cultural dimensions are primarily stable while specific perceptions and norms, particularly those related to the pandemic, can change rapidly. Our findings provide new evidence for theories of cultural change and have implications for policy, public health, daily life, and future trajectories of our societies.

## Introduction

Can cultures and their constituent norms, values, and perceptions, change rapidly in response to large-scale societal threats or is cultural change slow even in the face of such upheaval? And if cultural change does occur, in what way does it happen? We study this dual-sided question using data from two existing datasets that contain information on 29,761 individuals from 55 cities and 43 countries across the globe^1,2^. These data were collected recently before and soon after the emergence of COVID-19 and contain information on macro-cultural dimensions directly linked with established cultural theories, and specific perceptions, values, and norms. The relevant variables from these datasets that we study here have not been previously examined. In addition to a before and after comparison, we exploit variation in COVID-19 intensity across countries and use this to separate between three plausible mechanisms driving cultural changes: individual’s fear of COVID-19, their perceived prevalence of the disease, and the societal restrictions enacted by governments. This combination of large-scale before and after data, measures of culture on multiple dimensions, and the use of variation in disease severity, allows us to test key assumptions and propositions of multiple theories of cultural change in an unprecedented way.

Many theories agree that cultures tend to change slowly, with cultural change primarily happening across decades or generations^3–5^. Yet there are also examples of fast cultural change that are precipitated by sudden and external changes: the end of Apartheid in South Africa or the collapse of communist regimes in Eastern Europe and the resulting cultural shifts. Indeed, attitudes towards homosexuality and same sex marriage also display this fast change with support rapidly arising in recent years after decades of stability^4,6^.

There is also some evidence for fast cultural change in the context of natural and ecological disasters (e.g. floods, pandemics, earthquakes)^7–10^. Such cultural change may be relevant for public policy, including infection prevention behaviour, as prior research finds that culture is associated with public health behaviour such as smoking and alcohol use^11–13^ and COVID-19 prevention^14^. However, with few exceptions^2,15,16^ the existing research on the role of socio-ecological threats uses cross-sectional data and is typically limited by a lack of comparable pre-threat data e.g.^10,17–19^. Indeed, and specifically for the emergence of COVID-19, pre-pandemic data are often unavailable and even in the rare cases when they do exist, they were collected substantially before the emergence of COVID-19 making it unclear how comparable any changes are to after COVID-19’s emergence. The datasets we use avoid these issues. In fact, one of the main strengths of data we use is that the pre-pandemic data and those collected during the first months of the pandemic differ by only a few months making them comparable on most variables not affected by COVID-19.

In contrast to the uncertainty over the speed of cultural change, multiple theories from across disciplines, including sociology, psychology, and biology, make consistent and clear predictions. An increase in threat should lead to increases in collectivism, xenophobia/out-group hostility, norm-following and traditionalism. As individuals “clump together” to face the emergent threat, sharp distinctions are drawn between in-group and out-group and group members are expected to follow the cultural script.

COVID-19 provides an excellent case in which to study this multi-theory prediction. It is an extraordinary event during which countries across the world, with profound cultural, economic and social differences have found themselves sharing a health crisis where people—instead of boarding up windows, packing essentials, and evacuating to safer ground—have been asked to engage in social distancing, self-isolation, and quarantine if they had symptoms or test positive. In strikingly little time, myriad behaviours drastically changed including the time spent in the office, the adoption of social distancing in every interaction, the prohibition of public transport use, greetings, and even going out for non-essential activities. If cultural change can also happen in response to the disease, and not only behaviour, then we are likely to see this with the onset of the COVID-19 pandemic.

### Theory and evidence

Culture can be defined in many ways^20,21^. Our focus is specifically on what cultural sociologists call “personal declarative culture”^22^. This captures the aspects of culture that can be explicitly elicited such as values, attitudes, orientations, and worldviews, and contrasts with other aspects of culture, such as vocabularies, skills, dispositions, and associations.

#### Cultural change: only slow or also fast?

Theories of cultural dynamics typically posit that change happens slowly, primarily taking place over decades, centuries, or even millennia and often intergenerationally. Inglehart, for instance, writes that “basic values tend to change slowly through intergenerational population replacement, with multi-decade time-lags between the emergence of root causes and the time when cultural change becomes manifest in a society”^4^ (pp. 22, 23). Indeed, this is now well-established and extensive evidence supports the idea that cultural dynamics are often slow^4–6,23^.

Yet it also seems possible that, under certain conditions and situations, fast cultural change happens. Experiences of war have been found to shape religiosity after a few years in Uganda, Sierra Leone, and Tajikistan^24^. Terrorist events are found to affect outgroup hostility, political conservatism, and government support in a recent meta-analysis^9^ with many of the included papers studying short-term consequences including days, weeks, and months^25,26^. Winkler finds that exposure to natural disasters affects inferred social norm adherence^16^, while Szekely et al.^15^ use a 30-day online experiment to show that social norms of cooperation co-evolved with behaviour and this co-evolution was shaped by the risk of simulated collective risk.

Turning specifically to COVID-19, there are plausible reasons to think that this pandemic has led to cultural changes. It was an extremely destabilising event, breaking habits, behavioural patterns, and expectations. Crucially, this occurred within whole populations at a similar time creating a shared event, a “coordinated disturbance”, leading to a correlated upheaval of normal daily life. This may facilitate the emergence of new cultures since populations are facing uncertainty at the same time.

Indeed, recent studies find evidence for attitudinal, value, and norm change. A large-scale survey covering 58 countries and over 100,000 respondents, run between late March and early April 2020, found that social norms can arise quickly and effectively to support cooperation at a global scale^10^; a large-scale survey run in California and Rhode Islands, administered after about a month of stay-at-home orders, shows that American values, attitudes and activities (such as thinking about one’s own mortality, growing food, conserving resources) had changed dramatically during the COVID-19 pandemic^17^. While others find some evidence that authoritarian tendencies increased during COVID-19^18^ and so did anti-immigrant attitudes^19^. However, in almost all of the existing studies, data were collected only after the pandemic making it difficult to know the true change. Indeed, with before and after data, the evidence is much more mixed. Andrighetto and colleagues investigates the impact of the pandemic on tightness-looseness (the strength of cultural-level social norms and their enforcement)^27^ and other specific social norms from before COVID-19 to the early stage of the pandemic and find small changes in tightness-looseness and a substantial increase in handwashing norms^2^. While Drouhot and colleagues finds that support for immigrant and anti-discrimination norms against Asian origin population remain stable^28^. Finally, Vartanova and colleagues finds no change in the Americans’ endorsement of moral foundation after the onset of the COVID-19 pandemic^29^.

The contrast between short and fast culture change finds analogies with the separation between “active updating models”, under which people change their beliefs and attitudes in response to changing environments, discourses, and interactions, and “settled dispositions models”, in which people have durable dispositions that are acquired early in life^5^. Similarly, the differences can be addressed by contrasting a “gradualist model” for change and a “punctuated equilibrium” perspective that proposes that cultures change in jumps such that there are periods of stability punctuated by periods of rapid change^30^.

#### In what way can culture change?

If there is cultural change in response to COVID-19, then what kind of change can we expect? A range of theories, from sociology, psychology, and biology points in one direction. The Evolutionary Theory of Emancipation^31^, Evolutionary Modernization Theory^4^, Tightness-Looseness^27^, Parasite Stress Theory^3^ and more broadly the Behavioural Immune System^32^ predict that severe threat increases collectivism, duty, traditionalism, conformity and/or strength of social norms, and prejudice towards outgroup members. This is our core expectation. We now describe the theories that this is based on.

According to the “evolutionary theory of emancipation,” national populations’ subjective life orientations vary on a continuum from a “preventive closure” mentality, in which people emphasize uniformity, discipline, hierarchy, and authority, toward a “promotive openness” mentality, in which they emphasize the opposite traits, namely, diversity, creativity, liberty, and autonomy. The correspondence between objective living conditions and subjective life orientations consists in the fact that preventive closure is adaptive under pressing threats, while promotive openness is adaptive in the presence of promising opportunities.

Closely related, Evolutionary Modernization Theory^4^, building on classical modernization theory, argues that economic and physical insecurity are conducive to xenophobia, strong in-group solidarity, authoritarian politics and rigid adherence to their group’s traditional cultural norms, and conversely that secure conditions lead to greater tolerance of outgroups, openness to new ideas and more egalitarian social norms. The mechanism for this cultural and values change is based on emotional and experiential factors, such as whether people feel that survival is secure or insecure.

Tightness-Looseness proposes that cultures can be arranged according to the strength of their social norms and the extent to which they are enforced^27^. Tight cultures have strong norms that are heavily enforced while loose cultures have weak norms that are little enforced. It argues that societies that have experienced more ecological and social threats—frequent disease, warfare, and environmental catastrophes—throughout history develop tighter cultures to maintain order and survive chaos and crisis. In contrast, societies with less exposure to such ecological threats can afford to develop looser cultures that allow innovation and creativity at the cost of order. This proposition is well-supported by correlational evidence from cross-sectional surveys^27,33,34^. Recent work aims to get at the causality behind this claim. One online experiment finds that social norms of cooperation become stronger in response to increased risk of a simulated threat and weaken under lower risk conditions^15^. Similarly, survey evidence finds that earthquakes and epidemics increase people’s stated norm importance as well as the consistency of responses to questions in the European Social Survey^16^. However, in contrast to these studies Andrighetto and colleagues find a slight, albeit heterogeneous, decrease in tightness from before COVID-19 to the early stage of the pandemic. They also find large and consistent increases in the strength of handwashing social norms—a norm that is directly relevant to reducing the impact of the pandemic^2^.

The Behavioural Immune System theory posits that humans have evolved a suite of psychological and behavioural defences, in addition to physiological ones, that mitigate the threat posed by pathogens^19,32^. Specifically, it argues that behaviours toward potential pathogenic contaminators are elicited through the core emotion of disgust, and that an overgeneralization of such reactions may promote prejudicial behaviour and negative attitudes against those who look atypical or unfamiliar. Relatedly, Parasite Stress Theory claims that pathogen threats lead to psychological and cultural adaptations in terms of collectivism and outgroup prejudice^3,35^. There is cross-cultural evidence supporting these propositions^36^. This view is also linked to parenting and teaching, such that societies facing high pathogen stress are more likely to socialise children toward collectivist values centred on obedience, tolerance and interdependence^37^. In addition to this, it has been shown that high threat of disease generates xenophobia and low support for gender equality^3,35^.

Taking the existing theory and evidence together, our core expectation through this study is that *COVID-19 increases collectivism, duty, traditionalism, conformity, and outgroup prejudice.*

In addition to theoretical and empirical reasons for making this prediction, behavioural scientists and laypeople expect that COVID-19 changes culture in this way. Samples of behavioural scientists and lay Americans predicted that COVID-19 will increase explicit and implicit prejudice, traditionalism, and violence^38^. This is also reflected in some traditional and non-traditional popular media accounts^39–41^.

We operationalize our overarching expectation into a series of hypotheses (*Hypothesis 1* to *Hypothesis 7*; see Table 1). Prior findings^5^ and arguments^4,42^ suggest that cultural factors are more malleable the before people reach adulthood and change relatively little thereafter. If correct, we should find that changes in cultures is greater among younger people (*Hypothesis 8*). Inglehart calls this the socialisation hypothesis^4^.

**Table 1:** Key concepts, their operationalisations, motivation for their inclusion, and hypotheses.

Concept	Variable	Summary	Predictions
*Collectivism/individualism*^43^	Collectivism	Collectivism emphasizes low tolerance of deviation from in-group norms and a high attention on conformity and obedience	*Hypothesis 1:* Collectivism increases
*Duty/joy*^43^	Duty	Duty emphasizes hard work as important child quality and claim that people are in need because they are lazy	*Hypothesis 2:* Duty increases
*Traditionalism/autonomy* (World Values Survey)	Traditionalism	Autonomy emphasizes qualities like independence and determination/perseverance at the expense of religious faith and obedience	*Hypothesis 3:* Traditionalism increases
*Pro-fertility/individual choice norms*^6^	Individual choice	Pro-fertility norms support traditional gender roles and stigmatise non-traditional sexual behaviour	*Hypothesis 4*: Individual choice norms decrease
*Hygiene norms*^1^	Spitting inappropriateness	Where people think it is inappropriate to spit	*Hypothesis 5*: Spitting inappropriateness increases
*Perceived societal threats*^1^	Immigration	Immigration	*Hypothesis 6*: Immigration threat increases
	Subsistence	Food deprivation, lack of safe water, poor quality of air, diseases, and natural disasters	*Hypothesis 7*: Subsistence threat increases
*Socialization hypothesis*	Age differences	Intergenerational cultural change	*Hypothesis 8*: COVID-19 effect is larger on younger people

Notes: See Table S1 for wording of all items used.

## Methods

We combine data from a survey conducted between April-December 2019 (Wave 1)^1^ and the same survey conducted again after the onset of the COVID-19 pandemic, collected between April and June 2020 (Wave 2)^2^. We keep only respondents who are between 18 and 80 years old and who correctly passed an attention check placed at the end of the survey. The final sample contains responses from 43 countries and 55 cities (6 cities sampled only in Wave 1 while 1 city sampled only in Wave 2) reaching 29,761 valid respondents (15,275 in Wave 1 and 14,486 in Wave 2). 65% of respondents are female (19,489 out of 29,761), 75% (22,609 out of 29,761) are students, and the average age is 25 years old (s.d. = 9.70).

All participants gave their informed consent and we complied with all relevant ethical regulations. Approval of the study protocol was obtained from ethics committees and institutional review boards where required including for the University of Melbourne (Australia), Queen's University at Kingston (Canada), Universidad de los Andes (Colombia), Czech Academy of Sciences (Czech Republic), Universidad San Francisco de Quito (Ecuador), Monk Prayogshala (India), Trinity College Dublin (Ireland), Open University of Israel (Israel), LUISS University (Italy), United States International University—Africa (Kenya), Sunway University (Malaysia), University of Amsterdam (Netherlands), SWPS University of Social Sciences and Humanities (Poland), Universidade de Lisboa (Portugal), National University of Singapore (Singapore), University of Colombo (Sri Lanka), Koc University (Turkey), American University of Sharjah (United Arab Emirates), Brunel University London (United Kingdom), University of Kent (United Kingdom), University of South Carolina (United States of America), and New York University (United States of America). Ethical approval was not sought in countries where the approval received for the study conducted in Wave 1 was considered sufficient or where local legislation did not require ethical approval in the first place. The dataset generated and analysed during the current study are available at the persistent link placed at the end of the manuscript (“Data and materials availability”).

We study the following macro-cultural dimensions closely linked to theories that we study: (1) collectivism/individualism^43^, (2) duty/joy^43^, (3) autonomy/traditionalism (World Values Survey), (4) pro-fertility/individual choice norms^4,6^; and the following perceptions and norms: (5) perceived societal threats (immigration and subsistence factors)^1^ and (6) hygiene norms of spitting^1^. We adopt this broad approach, in which we study multiple distinct outcomes, in order to provide a high-level perspective on how COVID-19 shapes multiple different cultural aspects.

### Dependent variables and hypotheses

Most of our concepts are operationalised by following existing indices and then checked using exploratory factor analysis and Cronbach’s alpha for internal consistency. We start by creating individual-level outcomes. These have Cronbach’s alpha higher or equal than 0.65 and are normalised between 0 and 1. If individual level Cronbach’s alphas are below 0.65, we aggregate the outcomes to the country-level and that have sufficient consistency. We specify the hypotheses for all of our measures below which are based on our general framework. We also include a number of hypotheses for specific perceptions and norms that are not derived from theory but closely drawn from the implications of the pandemic (e.g. fearing disasters more after the emergence of COVID-19). We do not have specific hypotheses for a number of measures but test their change in an exploratory way and include the results in the Supplementary Materials. Table S1 in the Supplementary Materials contains the wording of all items used. Our measures and their associated hypotheses are summarised in Table 1.

The macro-cultural variables of collectivism/individualism, duty/joy, and traditionalism/autonomy are created from subjects’ responses to a multiple response question that asks subjects which “qualities children should be encouraged to learn at home” (question drawn from the World Values Survey). Respondents can choose up to five responses from a list of 10 different qualities that includes religious faith, obedience, and feelings of responsibility.

#### Collectivism/individualism

This dimension represents the importance that people place on their relationship to their collectives^43^. Collectivist cultures place a high value on the relationships among group members and people are expected to follow their group’s norms and obligations. In contrast, individualist societies depend more on impartial institutions and people follow universal norms, while towards the in-group there are weaker expectations of conformity and norm-breaking is more tolerated. We follow the operationalisation of Beugelsdijk and Welzel^43^ such that Collectivism = Religious faith + Obedience−Feeling of responsibility, where the qualities are coded 1 if selected, 0 otherwise. People with high individualist values tend to feel that religion is not important, that responsibility is an important quality in children, and that it is important to be successful. Conversely, collectivist people think that religious faith and obedience are important. We study this at the country level (Cronbach’s alpha = 0.68).

#### Duty/joy

People who emphasize duty score high on the importance of hard work as an important quality in children and in their response to questions about people who are in need because they are lazy. Instead, people who emphasize joy tend to live in bigger cities, do not find a good income important in a job, embrace democracy, and find imagination an important child quality^43^. We have data on the importance of hard work as an important quality in children, as well as imagination and follow Beugelsdijk and Welzel^43^ and calculate Duty = Hard work−imagination. We study this at the country level (Cronbach’s alpha = 0.65).

#### Traditionalism/autonomy

People who emphasize traditionalism score high on importance of religious faith and obedience over independence and determination. We create the index following the World Values Survey (WVS) and calculate it with the formula Traditionalism = Religious faith−Obedience—Independence—Determination. We study this at the country level (Cronbach’s alpha = 0.65).

#### Pro-fertility/individual choices norms

This dimensions is derived from Inglehart et al.^6^ and has been argued to be a key consequence of modernisation. Pro-fertility norms emphasise traditional gender roles and stigmatise sexual behaviour not linked to reproduction while the latter support gender equality and tolerance of non-traditional sexual behaviour. Put differently, they deal with aspects related to gender equality, divorce, abortion, homosexuality, and suicide. We built an additive index using 4 items (question text taken from the World Values Survey). This asks respondents how justified are homosexuality, divorce, abortion, and suicide. We study the measure at the individual level since it has high internal validity (Cronbach’s alpha = 0.81) and exploratory factor analysis supports the proposed grouping.

#### Hygiene norms

These are measured with a multiple response question that asks about where respondents think it is inappropriate to spit: in the kitchen sink, on the sidewalk, on the kitchen floor, on the soccer field, in the water in a public swimming pool and in the forest. We create a single measure based on those items and we study it at the individual level since it has sufficient internal consistency (Cronbach’s alpha = 0.65).

#### Perceived societal threats

This is measured through a multiple responses question that includes nine possible responses about perceptions of real threats from^1^. We study two constructs: one item alone of immigration as a societal threat and perceived subsistence threats that combine food deprivation, lack of safe water, poor quality of air, diseases, natural disasters. We study the measures at the individual level since the former relies on only one item and the latter has high internal validity (Cronbach’s alpha = 0.72) and exploratory factor analysis supports the proposed grouping of subsistence threat.

Based on our general theoretical framework we formulate the following hypotheses: collectivism increases (*Hypothesis 1*), duty increases (*Hypothesis 2*), traditionalism increases (*Hypothesis 3*), individual choice norms decrease (*Hypothesis 4*), hygiene norms prohibiting spitting increase (*Hypothesis 5*), and immigration threat perception increases (*Hypothesis 6*), and after the emergence of COVID-19. Even though we do not rely on specific theoretical assumptions we anticipate—directly based on the response to COVID-19—that subsistence threat perception increases (*Hypothesis 7*).

#### Socialisation hypothesis

Finally, following the socialisation hypothesis, we test whether participants’ age would influence our outcome variables. We expect to find differences between young and old, such that the changes in the outcomes (before and after COVID-19) are larger for younger people than for older people (*Hypothesis 8*).

Finally, our dataset also contains a number of questions that we do not focus on in the main text because (1) the questions are not directly linked to the theories that we test or (2) they display low internal validity. Instead, we study them in exploratory way and report the results in the Supplementary Materials. These questions are: Hofstede’s cultural dimensions (collectivism/individualism, restraint/indulgence, and power distance); justifiability of interpersonal violence; conflict (between countries and within country) and overpopulation as perceived societal threat; and hygiene norms of brushing teeth. Each concept is described in terms of dimensions used, wording, and interpretation in the Supplementary Materials (Table S16). Although Hofstede was the first to quantify cultural orientations held by people in more than 60 countries and his framework has been extensively used, Hofstede’s data collection procedure and sample have been criticized see also^44^. For this reason, we do not rely on his operationalisations in the main text. Nevertheless, due to the theoretical importance and widespread empirical use, we report these results in the Supplementary Materials.

### Statistical approach

We proceed in two steps. First, we analyse changes in the outcomes between Wave 1 and Wave 2 using multilevel models with random intercepts at the country (*c*), city (*k*) and individual (*i*) levels and cluster-robust standard errors at the country-level:

$$Y_{cki} = \, \beta_{0c} + \, \beta_{0k} + \, \beta_{1} Wave2_{cki} + \, \delta Z_{cki} + \, e_{cki}$$

when the outcome of interest is individual-level (pro-fertility/individual choices norms, perceived societal threats, hygiene norms). For country-level outcomes (collectivism/individualism, duty/joy, traditionalism/autonomy), we use OLS linear regression models with heteroskedastic robust standard errors:

$$Y_{c} = \, \beta_{0c} + \, \beta_{1} Wave2_{c} + \, \delta Z_{c} + \, e_{c}$$

where *Y* is the vector of the outcome variables, *Wave2* is a dummy variable that assumes the value 1 if the observation is in Wave 2 and 0 if the observation is in Wave 1; and *Z* is a vector of control variables, that are being student/no student, age, and gender, to account for potential sample variation between Wave 1 and Wave 2. We also consider deaths and cases which account for the differences in severity of COVID-19 across countries. To test the socialisation hypothesis (*Hypothesis 8*) we add an interaction effect between *Wave2* and age to the relevant individual-level or country-level analyses. We also conduct analyses with fixed-effects for each country and cluster-robust standard errors at the country-level as an alternative modelling strategy.

The above analyses identify whether there were changes in the outcome variables between Wave 1 and Wave 2. It provides initial indications that COVID-19 causally changes the outcome variables, since apart from the emergence of COVID-19 and associated implications, there were few systematic large-scale events, none comparable to the COVID-19 pandemic, across the 43 countries during the time period of our study. Nevertheless, to further reduce the possibility of confounders and to identify the specific mechanisms of COVID-19 that led to any observed changes, we undertake a second step.

Second, for those outcomes that show statistically significant changes, we exploit variation in COVID-19 intensity using a similar approach to differences-in-difference analyses and study mechanisms underlying the changes. We use three variables that capture the strength of different aspects of COVID-19. These are: (1) the subject’s fear of the COVID-19 pandemic (index built with three items that ask the subject how concerned he/she is by the spread of the COVID-19, how much fear does he/she has by the spread of the COVID-19, and how dangerous does he/think COVID-19 is; Cronbach’s alpha = 0.84), (2) COVID-19 perceived prevalence (What percent of people living in your province do you think have been infected with COVID-19?), and (3) government stringency, used to capture the degree to which countries’ governments implemented strict (or lenient) anti-disease policies. Government stringency uses the Stringency Index subset from the Oxford COVID-19 Government Response Tracker^45^. We also check whether deaths and cases, which account for the differences in severity of COVID-19 across countries, affect our results and find that they do not.

Using these variables allows us to exploit variation in COVID-19 intensity in an approach similar to a differences-in-difference analysis that is also used in existing studies^7,46^. Crucially, as additional variables are unlikely to be correlated with both the predictors of fear, prevalence, and government stringency and the change in the outcomes, we are able to more rigorously test the causal effect of the pandemic. The primary differences to differences-in-differences analysis are that, instead of a dichotomous separation between “treated” and “untreated” groups, we have groups (countries) that have received different levels of treatment and there is no entirely untreated group. This latter point is likely to reduce the estimated effect of the mechanisms. A key strength of our data is that we can exploit variation in COVID-19 intensity that we measure closely with variables collected in our questionnaire. Moreover, since the time gap between the end of Wave 1 and start of Wave 2 data collection is reasonably short (around four months), we can be confident that time varying factors such as changes in healthcare infrastructure spending or GDP growth, which could cause problems of endogeneity, do not play a role.

The specific models we use in the second stage, to investigate the mechanisms of changes, have country-level observations and OLS linear regression models with heteroskedastic robust standard errors:

$$\Delta Y_{c} = \, \beta_{0c} + \, \beta_{1} Fear_{c} + \, \beta_{2} Perc.Preval_{c} + \, \beta_{3} Stringency_{c} + \, \delta Z_{c} + \, e_{c}$$

where *ΔY* is the difference in mean between Wave 2 and Wave 1 for the outcome variables; *Fear* is the variable measuring fear of COVID-19; *Perc.Preval* is the variable measuring the perceived prevalence of cases; *Stringency* is the government stringency policy variable; and *Z* is the vector of control variables (proportion student/no student status and female, and mean age).

We also performed sensitivity power analysis to understand if we have sufficient achieved power in our models testing the change in waves in our dependent variables. Sensitivity power analysis allows the researcher to determine the minimum effect size that the study was sensitive to for a certain level of power, based on the sample size and the alpha level specified. Given a sample of 29,761 individuals, a significance level of alpha = 0.05 and a desired power of 0.80, we estimate that a minimum detectable effect size f^2^ that could be detected are tiny and equal to f^2^ < 0.001 (two sided). Instead, at a country level, given a sample of 43 countries, a significance level of alpha = 0.05, and a desired power 0.80, we estimate the minimum detectable effect size f^2^ = 0.2 (two sided).

## Results

**Figure 1:**
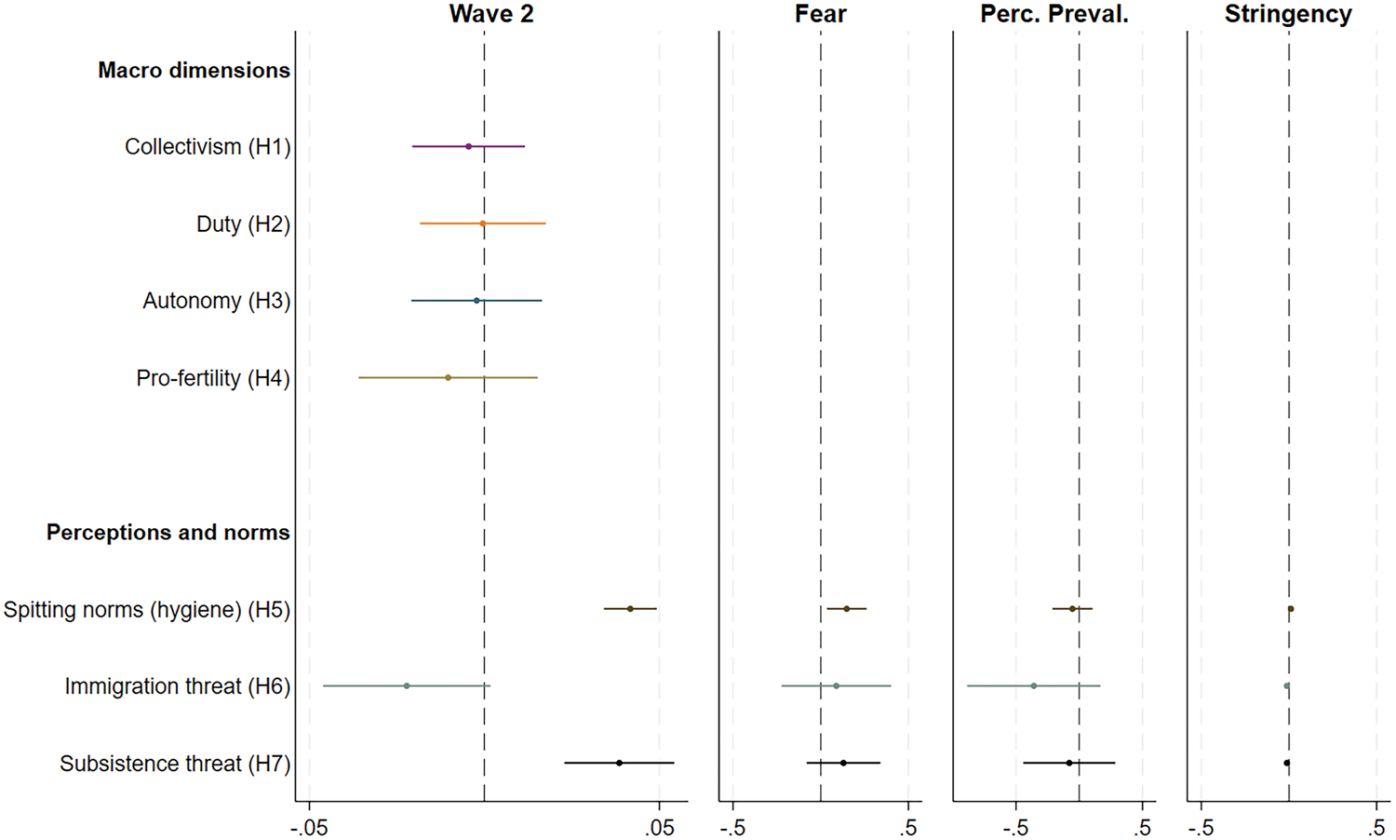
Model estimates of the Wave 1 to Wave 2 change and the mechanisms driving the change. Mechanism coefficients are only plotted for coefficients that change significantly and are robust.

We report the results of the first step (i.e., change from Wave 1 to Wave 2) of our analysis for all the outcomes studied. Instead, concerning the second step (i.e., mechanisms underlying the change), we report only the results for those outcomes that show statistically significant change in the first step. Figure 1 summarizes the results. It shows the coefficient estimates of the change from Wave 1 to Wave 2 (Wave 2) and the mechanisms driving the change (Fear, Perc.Preval., Stringency).

### Collectivism/individualism

In contrast with Hypothesis 1, collectivism does not change from Wave 1 to Wave 2 (β_Wave2_ = − 0.004, 95% CI = [− 0.020; 0.011], *p* = 0.575, Table S3) in a statistically significant way.

### Duty/joy

In contrast with Hypothesis 2, duty does not change from Wave 1 to Wave 2 (β_Wave2_ = − 0.0004, 95% CI = [− 0.018; 0.017], *p* = 0.961, Table S4) in a statistically significant way.

### Traditionalism/autonomy

In contrast with Hypothesis 3, traditionalism does not change from Wave 1 to Wave 2 (β_Wave2_ = − 0.002, 95% CI = [− 0.020; 0.016], *p* = 0.813, Table S5) in a statistically significant way.

### Pro-fertility/individual-choice norms

In contrast with Hypothesis 4, individual choice norms do not change from Wave 1 to Wave 2 (β_Wave2_ = − 0.010, 95% CI = [− 0.016; − 0.005], *p* < 0.428, Table S6).

### Hygiene norms

Consistent with Hypothesis 5, inappropriateness of spitting increase (β_Wave2_ = 0.042, 95% CI = [0.034; 0.049], *p* < 0.001, Table S9), with an increase in absolute terms from 0.617 in Wave 1 to 0.664 in Wave 2 (Cohen’s *d* = 0.177), and fear of COVID-19 appears to be the main mechanism that drives the change (β_Fear_ = 0.148, 95% CI = [0.035; 0.261], p = 0.012, Table S13); also government stringency drives the change but the effect is low (β_Gov.Str._ = 0.011, 95% CI = [0.003; 0.018], p = 0.007, Table S13).

### Perceived societal threats

Contrary to Hypothesis 6, perception of immigration as a real threat to society decreases (β_Wave2_ = − 0.022, 95% CI = [− 0.046; -0.001], *p* = 0.069, Table S7, with a small decrease in absolute terms from 0.286 in Wave 1 to 0.283 in Wave 2 (Cohen’s *d* = 0.007). While, and consistent with Hypothesis 7, subsistence threat increases (β_Wave2_ = 0.039, 95% CI = [0.022; 0.054], *p* < 0.001, Table S8), with an increase in absolute terms from 0.478 in Wave 1 to 0.516 in Wave 2 (Cohen’s *d* = 0.10). This change in subsistence index is driven by the item that correspond to the perception of diseases as societal threat, which displays a large increase from 0.540 in Wave 1 to 0.698 in Wave 2 (Cohen’s *d* = 0.329). Concerning the mechanisms, we find no statistically significant evidence for any of those (for immigration as societal threat *p*-values vary between 0.565 for fear of COVID-19, 0.174 for perceived prevalence of cases and 0.169 for government stringency, Table S11; for subsistence threat *p*-values vary between 0.217 for fear of COVID-19, 0.663 for perceived prevalence of cases and 0.175 for government stringency, Table S12).

### Socialisation hypothesis

We find some support for Hypothesis 8: pro-fertility norms, subsistence threats and hygiene norms of spitting display a statistically significant effect (*p*-values equal to 0.004 for subsistence threat perception; equal to 0.024 for pro-fertility norms, and equal to 0.018 for hygiene norms of spitting), although the effect is always small (in every case the β = 0.001). While we find non-statistically significant associations for collectivism, duty, autonomy and immigration threat.

Table S14 shows the results of the additional analysis with OLS fixed-effects analyses with clustered robust standard errors. We find no substantive changes. Subsistence threat changes and spitting norms change, remain highly significant. Immigration threat change becomes significant at the 10%-level and pro-fertility norm change becomes non-significant.

A potential reason why many of our outcomes show no average change from Wave 1 to Wave 2 could be because the change is moderated by trust in the political system and the health system. Specifically, one may expect that people who do not trust politics or the health system should decrease in collectivism, duty, and autonomy, while those high in trust should increase in these variables. If this were to be the case, one would expect an increase in the distribution of these measures across the waves. We test for this and find little evidence for changes in distributions (see Table S15 and Fig. S5).

## Discussion

Our paper studies the effect that COVID-19 had on culture in the short-term. We asked two questions: are there changes in personal declarative culture? If so, in what way? Our expectations for how culture would change drew on a large body of theoretical and empirical literature and predicted an increase in collectivism, duty, traditionalism, conformity (pro-fertility norms), and outgroup prejudice.

Contrary to Hypothesis 1, 2, 3, and 4 collectivism, duty, traditionalism, and individual choice norms did not change. In line with Hypothesis 5, hygiene norms prescribing spitting increased. Concerning perceived societal threats, immigration decreased, in contrast with Hypothesis 6, while, and consistent with Hypothesis 7, perceived subsistence threats increased.

Regarding mechanisms, we only found that the increase in hygiene norms of spitting is related to variation in threat perceptions, so that those who fear more about the COVID-19 pandemic have changed their hygiene norms of spitting more. These norms are particularly revealing since they regulate behaviour that harms others but not the actor. In contrast to, e.g. keeping to well-ventilated areas, washing hands, or avoiding crowded areas, which are actions that can benefit both the person taking the action and others around them, and can be thus taken for prosocial or individualistic motivations, refraining from spitting cannot be taken for individualistic motives. This implies that if norms restricting spitting increase, then this is good evidence for an increase in other-helping hygiene norms.

Concerning the socialisation hypothesis (Hypothesis 8), even though we find a statistically significant effect for some outcomes, the effects are small. Thus substantively, we also find little support for this hypothesis.

By putting all of these results together, we can see an overall outline of our findings. Our macro-cultural dimensions (collectivism, duty, traditionalism, and individual choice norms) primarily display stability and thus do not support the theories we started with. In contrast, perceptions and norms (e.g. spitting social norms and perceived threat), particularly those relevant to the disease change clearly and substantially.

To conclude, what do our findings mean? They suggest that even a shock as substantial and large as COVID-19 was, at least in the short-term, insufficient to create changes in our macro-cultural dimensions. In contrast, the specific norms and perceptions considered can change rapidly and in response to threats.

What are the implications of our findings? Our results provide evidence of an important dynamic of cultural change following an external shock namely that only specific norms, particularly those related to the pandemic, change rapidly in response to the threat. Concerning the theoretical framework that we started with: we find little support of it in our results. Yet our study examines short term changes. What would happen in the long run remains to be seen and may display results consistent with those theories. Further work is needed to complement the understanding of the specific mechanisms underlying cultural dynamics.

Finally, there is substantial between-country heterogeneity in the changes. Some countries changed very little while others changed substantially. Understanding this heterogeneity is an important next step to take; part of it is likely to be explained by countries’ cultural context and their structural conditions (i.e., GPD, health care system, etc.).

### Supplementary Information

Supplementary Information.

## Data Availability

The survey and the analysis code are available at the Open Science Framework: 10.17605/OSF.IO/2YUQS.
