# Author Correction: Evidence from 43 countries that disease leaves cultures unchanged in the short-term

**DOI:** 10.1038/s41598-024-63295-2

**Published:** 2024-06-03

**Authors:** Gian Luca Pasin, Aron Szekely, Kimmo Eriksson, Andrea Guido, Eugenia Polizzi di Sorrentino, Giulia Andrighetto

**Affiliations:** 1 Department of Social and Political Sciences, University of Milan, Milan, Italy; 2 Collegio Carlo Alberto, Turin, Italy; 3 Institute of Cognitive Sciences and Technologies, National Research Council of Italy, Rome, Italy; 4 Center for Cultural Evolution, Stockholm University, Stockholm, Sweden; 5 CEREN EA 7477, Burgundy School of Business, Université Bourgogne Franche-Comté, Dijon, France; 6 Institute for Futures Studies, Stockholm, Sweden; 7 Malardalens University, Västerås, Sweden

Correction to: *Scientific Reports* 10.1038/s41598-023-33155-6, published online 18 March 2024.

The original version of this Article contained errors.

In the original version of this Article, Figure 1 contained errors in the Y axis, where the criteria order was incorrect. The incorrect Figure and its accompanying legend appear below.

**Figure 1:**
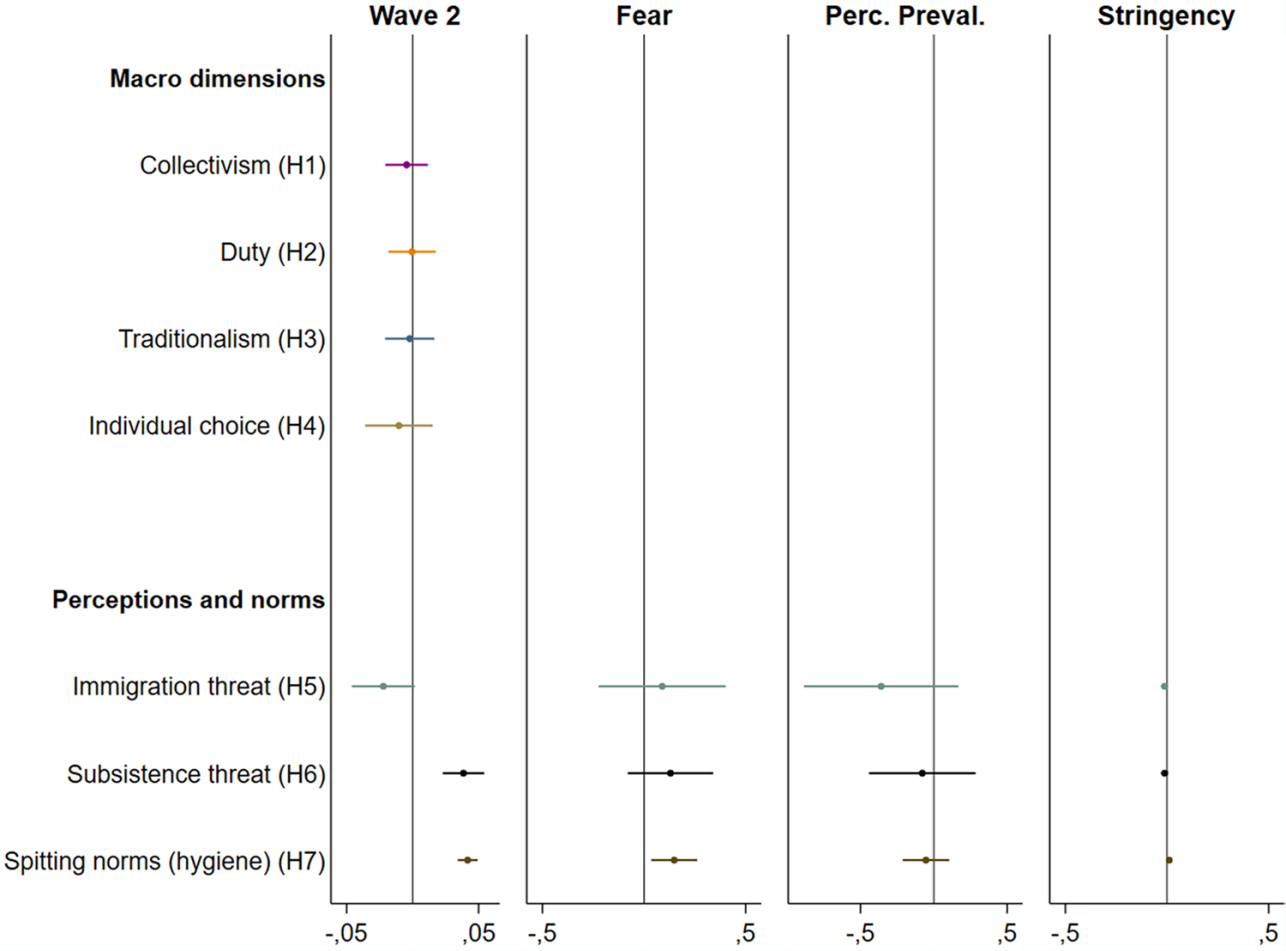
Model estimates of the Wave 1 to Wave 2 change and the mechanisms driving the change. Mechanism coefficients are only plotted for coefficients that change significantly and are robust.

Furthermore, in the Abstract section

“We derive specific hypotheses from this prediction and use survey data from 29,761 respondents, in 55 cities and 43 countries, collected before (April–December 2019) and recently after the emergence of COVID-19 (April–June 2020) to test them.”

now reads,

“We derive specific hypotheses from this prediction and use survey data from 29,761 respondents, in 55 cities and 43 countries, collected before (April–December 2019) and recently after the emergence of COVID-19 (March–July 2020) to test them.”

in the Discussion section, where

“Our results provide evidence of an important dynamic of cultural change following an external shock such that specific social norms change in response to the threat.”

now reads,

“Our results provide evidence of an important dynamic of cultural change following an external shock namely that only specific norms, particularly those related to the pandemic, change rapidly in response to the threat”.

In the Methods section, under the subsection ‘Statistical approach’

“Instead, at a country level, given a sample of 41 countries, a significance level of alpha = 0.05, and a desired power 0.80, we estimate the minimum detectable effect size f^2^ = 0.2 (two-sided).”

now reads,

“Instead, at a country level, given a sample of 43 countries, a significance level of alpha = 0.05, and a desired power 0.80, we estimate the minimum detectable effect size f^2^ = 0.2 (two-sided).”

where

“*Fear* is the variable measuring fear of COVID-19; *Prevalence* is the variable measuring the perceived prevalence of cases; *GovStr* is government stringency policy variable; and *Z* is the vector of control variables (proportion student/no student status and female, and mean age).”

now reads,

“*Fear* is the variable measuring fear of COVID-19; *Perc.Preval* is the variable measuring the perceived prevalence of cases; *Stringency* is the government stringency policy variable; and *Z* is the vector of control variables (proportion student/no student status and female, and mean age).”

and where

“People who emphasize duty score high on the importance of hard work as an important quality in children and in their response to question about people are in need because they are lazy^5^. Instead, people who emphasize joy tend to live in bigger cities, do not find a good income important in a job, embrace democracy, and find imagination an important child quality Welzel^43^.”

now reads,

“People who emphasize duty score high on the importance of hard work as an important quality in children and in their response to questions about people who are in need because they are lazy. Instead, people who emphasize joy tend to live in bigger cities, do not find a good income important in a job, embrace democracy, and find imagination an important child quality^43^. We have data on the importance of hard work as an important quality in children, as well as imagination and follow Beugelsdijk and Welzel^43^ and calculate Duty = Hard work-imagination. We study this at the country level (Cronbach’s alpha = 0.65).”

Moreover, in the Data availability section, where

“All data, code, and materials will be made publicly available on the OSF. They are currently available for review at: https://www.dropbox.com/sh/3w444u060nvfp6g/AAAi8bLJNlXQ0T5LY5jJpul3a?dl=0.”

now reads,

“The survey and the analysis code are available at the Open Science Framework: 10.17605/OSF.IO/2YUQS.”

Finally, Reference 2 was incorrect.

Reference 2

“Andrighetto, G. et al. Changes in social norms during the early stages of the Covid-19 pandemic across 43 countries. Cond. Accept Nat. Commun. 2022, 18 (2022).”

now reads,

Reference 2

“Andrighetto, G. et al. Changes in social norms during the early stages of the COVID-19 pandemic across 43 countries. Nat. Commun. 15, 1436 (2024).”

The original Article has been corrected.

