# Supplementary material for: Evidence from 43 countries that disease leaves cultures unchanged in the short-term
Source: Sci Rep. 2024 Mar 18;14:6502. doi: 10.1038/s41598-023-33155-6 (PMC10948757; doi:10.1038/s41598-023-33155-6)
Supplement: Supplementary file 1 — Supplementary Information. [file 41598_2023_33155_MOESM1_ESM.docx]

Supplementary Materials for

**Evidence from 43 countries that disease leaves cultures unchanged in the short-term**

**Authors:** Gian Luca Pasin^1,2,3*^, Aron Szekely^2,3^, Kimmo Eriksson^4^, Andrea Guido^3,5^, Eugenia Polizzi di Sorrentino^3^, Giulia Andrighetto^3,6,7^

**Affiliations**:

^1^Department of Social and Political Sciences, University of Milan; Milan, Italy

^2^Collegio Carlo Alberto; Turin, Italy.

^3^Institute of Cognitive Sciences and Technologies, National Research Council of Italy; Rome, Italy.

^4^Center for Cultural Evolution, Stockholm University; Stockholm, Sweden.

^5^CEREN EA 7477, Burgundy School of Business, Université Bourgogne Franche-Comté; Dijon, France.

^6^Institute for Futures Studies; Stockholm, Sweden.

^7^Malardalens University; Vasteras, Sweden.

*Corresponding author. Email: [gianluca.pasin@unimi.it](mailto:gianluca.pasin@unimi.it)

**Materials**

Sample

Our sample includes data collected in two waves, one related to the period before the breakout of the pandemic (April-December 2019, Wave 1) and one to the period during the first pandemic breakout (March-July 2020, Wave 2).

For comparability of samples across waves and among countries, we set out to collect data from approximately 200 college students at least in a major city in each country, which was achieved in all countries (Table S1). To assess the robustness of the country-level measures obtained from these samples, we complemented the main sampling strategy by collecting additional data from non-student samples. In our analyses, we considered subjects who correctly passed an attention check placed at the end of the survey (i.e., subjects had to click a specific item response). The total number of discarded observations because of failed attention check is minimal across waves relative to our sample size (197 in Wave 1, 202 in Wave 2). We additionally excluded subjects who declared an age under 18 (157 in Wave 1, 222 in Wave 2).

The final database includes responses from 43 countries, 55 locations (6 of which sampled only in wave 1, while only 1 sampled exclusively in wave 2), and 29760 valid respondents (see Table S2).

Survey administering

The survey was translated into 30 different languages, following the usual practice of independent translation and back-translation. The study was conducted anonymously online using Qualtrics.

IRB approval

All participants gave their informed consent and we complied with all relevant ethical regulations. Approval of the study protocol was obtained from ethics committees and institutional review boards where required.

**Table S1.** Concepts, dimensions, wording and interpretation of all items used.

| **Concept** | **Dimensions** | **Wording** | **Interpretation** |
| --- | --- | --- | --- |
| *Collectivism/ individualism*^[1]^ | Collectivism/individualism | Here is a list of qualities that children can be encouraged to learn at home. Which, if any, do you consider to be especially important? Please choose up to five!   - Religious faith - Obedience - Feeling of responsibility | Low values: individualism  High values: collectivism |
| *Duty/joy*^[1]^ | Duty/joy | Here is a list of qualities that children can be encouraged to learn at home. Which, if any, do you consider to be especially important? Please choose up to five!   - Hard work - Imagination | Low values: joy  High values: duty |
| *Autonomy index* (World Values Survey) | *Traditionalism/autonomy* | Here is a list of qualities that children can be encouraged to learn at home. Which, if any, do you consider to be especially important? Please choose up to five!   - Religious faith - Obedience - Independence - Determination | Low values: traditionalism  High values: autonomy |
| *Pro-fertility/*  *individual choice norm*^[2]^ |  | Please tell us for each of the following actions whether you think it can always be justified, never be justified, or something in between   - Homosexuality - Divorce - Abortion - Suicide | Low values: individual choice norms  High values: pro-fertility norms |
|  |  | Which of the following do you think of as real threats to your society (tick all  that apply) |  |
| *Perceived societal threats*^[3]^  *Hygiene norms*^[3]^ | Immigration threat | - Immigration | Low values: less perception as real threat.  High values: more perception as real threat. |
|  | Subsistence threat | - Food deprivation - Lack of save water - Poor quality of air - Natural disasters - Diseases |  |
|  | Spitting inappropriateness | Where do you think it is not appropriate for people to spit? Tick any that apply   - In the kitchen sink - On the sidewalk - On the kitchen floor - On the soccer field - In the water in a public swimming pool - In the forest | Low values: low hygiene  High values: high hygiene |

| **Table S2.** Descriptive statistics: % of female and students in our samples, and average age by country. | | | | | | | | | |
| --- | --- | --- | --- | --- | --- | --- | --- | --- | --- |
| *Country* | *City* | *N* | | *% Female* | | *% Students* | | *Age* | |
|  |  | *Wave 1* | *Wave 2* | *Wave 1* | *Wave 2* | *Wave 1* | *Wave 2* | *Wave 1* | *Wave 2* |
| ARE | Sharjah | 306 | 285 | 64.47 | 65.26 | 100.00 | 100.00 | 20.02 | 19.91 |
| ARG | Cordoba | 448 | 236 | 68.92 | 84.68 | 47.77 | 26.07 | 25.84 | 49.87 |
| ARM | Yerevan | 343 | 371 | 66.76 | 61.85 | 66.47 | 64.96 | 24.01 | 24.48 |
| AUS | Melbourne | 249 | 183 | 75.81 | 81.32 | 100.00 | 100.00 | 19.65 | 19.70 |
| BIH | Banja Luka | 236 | 169 | 50.42 | 65.87 | 100.00 | 100.00 | 21.19 | 22.66 |
| BRA | Sao Paolo | 283 | 230 | 55.36 | 64.35 | 68.55 | 40.00 | 31.34 | 40.01 |
| CAN | Kingston | 205 | 459 | 55.61 | 55.82 | 100.00 | 100.00 | 19.79 | 19.84 |
| CAN | Toronto | 222 | NA | 78.18 | NA | 100.00 | NA | 20.77 | NA |
| CHL | Santiago | 129 | 116 | 42.97 | 43.10 | 100.00 | 100.00 | 20.90 | 21.03 |
| CHN | Beijing | 398 | NA | 71.36 | NA | 71.61 | NA | 22.81 | NA |
| CHN | Guangzhou | 301 | 88 | 68.37 | 56.82 | 70.10 | 100.00 | 24.46 | 19.95 |
| CHN | Shenzhen | 327 | 375 | 71.06 | 39.00 | 100.00 | 100.00 | 20.16 | 20.42 |
| COL | Bogota | 342 | 896 | 60.41 | 59.53 | 67.25 | 70.09 | 25.62 | 24.89 |
| CZE | Brno | 378 | 446 | 77.01 | 76.42 | 68.78 | 64.57 | 28.13 | 30.26 |
| DEU | Cologne | 296 | 202 | 57.44 | 69.15 | 32.09 | 100.00 | 25.60 | 29.06 |
| DEU | Hagen | 369 | 226 | 78.36 | 73.64 | 0.00 | 0.00 | 31.28 | 31.41 |
| ECU | Quito | 285 | 247 | 61.97 | 62.60 | 84.56 | 86.64 | 22.65 | 23.21 |
| ESP | Madrid | 322 | 429 | 43.44 | 49.06 | 60.87 | 58.97 | 28.30 | 31.17 |
| EST | Tallin | 288 | 253 | 74.91 | 69.44 | 77.43 | 49.01 | 30.78 | 33.80 |
| FIN | Helsinki | 251 | 364 | 81.47 | 82.99 | 89.64 | 81.32 | 30.64 | 32.55 |
| GBR | Canterbury | 315 | 79 | 82.22 | 78.21 | 60.95 | 100.00 | 26.20 | 21.03 |
| GBR | London | 109 | 314 | 88.99 | 64.97 | 100.00 | 35.99 | 19.13 | 30.26 |
| GRC | Athens | 163 | NA | 55.35 | NA | 90.18 | NA | 27.16 | NA |
| GRC | Patra | 343 | 523 | 75.07 | 81.24 | 65.31 | 69.60 | 27.36 | 25.64 |
| HUN | Budapest | 452 | 840 | 79.73 | 83.83 | 100.00 | 60.83 | 24.47 | 29.02 |
| IDN | Surabaya | 314 | 391 | 80.77 | 74.61 | 69.11 | 65.22 | 22.55 | 23.16 |
| IND | Mumbai | 265 | 223 | 87.64 | 57.40 | 95.47 | 100.00 | 19.71 | 22.21 |
| IRL | Dublin | 239 | 146 | 57.98 | 53.47 | 19.25 | 100.00 | 22.15 | 21.77 |
| ISL | Reykjavik | 478 | 305 | 76.82 | 79.40 | 78.66 | 76.39 | 30.66 | 31.17 |
| ISR | Raanana | 335 | 349 | 60.18 | 67.72 | 67.46 | 60.17 | 27.82 | 28.25 |
| ITA | Rome | 186 | 279 | 44.26 | 51.26 | 100.00 | 100.00 | 22.25 | 24.40 |
| ITA | Turin | 206 | 278 | 72.14 | 64.86 | 100.00 | 100.00 | 23.44 | 24.60 |
| JPN | Hikone | 306 | NA | 60.98 | NA | 100.00 | NA | 19.67 | NA |
| JPN | Kanagawa | 258 | NA | 43.87 | NA | 100.00 | NA | 20.14 | NA |
| JPN | Tokyo | NA | 472 | NA | 39.07 | NA | 100.00 | NA | 18.97 |
| KEN | Nairobi | 190 | 115 | 48.68 | 75.44 | 100.00 | 100.00 | 22.02 | 27.77 |
| KOR | Seoul | 370 | 499 | 54.70 | 57.66 | 66.22 | 57.11 | 27.16 | 29.48 |
| LKA | Colombo | 191 | 169 | 61.90 | 51.50 | 100.00 | 100.00 | 23.18 | 23.15 |
| MYS | Kuala Lumpur | 347 | 160 | 52.80 | 69.43 | 69.74 | 85.00 | 25.07 | 22.01 |
| NGA | Nsukka | 302 | 396 | 56.67 | 57.25 | 60.93 | 70.45 | 27.95 | 25.04 |
| NLD | Amsterdam | 273 | 346 | 54.58 | 68.02 | 100.00 | 100.00 | 21.78 | 21.81 |
| PER | Lima | 272 | 447 | 69.26 | 54.36 | 43.38 | 48.77 | 33.56 | 27.91 |
| POL | Warsaw | 542 | 417 | 70.79 | 69.88 | 43.91 | 45.56 | 35.02 | 35.29 |
| PRT | Lisbon | 140 | 158 | 88.57 | 74.68 | 100.00 | 100.00 | 24.31 | 25.68 |
| RUS | Moscow | 266 | 226 | 77.86 | 87.39 | 100.00 | 100.00 | 23.03 | 20.68 |
| RUS | St Petersburg | 121 | 16 | 74.79 | 100.00 | 100.00 | 100.00 | 21.88 | 24.50 |
| SAU | Riyhad | 317 | 140 | 27.62 | 43.48 | 68.77 | 76.43 | 24.65 | 25.06 |
| SGP | Singapore | 202 | 102 | 68.66 | 56.86 | 100.00 | 100.00 | 21.95 | 21.43 |
| SWE | Linkoping | 163 | NA | 54.32 | NA | 100.00 | NA | 24.75 | NA |
| SWE | Stockholm | 40 | NA | 51.28 | NA | 100.00 | NA | 37.38 | NA |
| TUR | Istanbul | 229 | 232 | 83.41 | 71.98 | 100.00 | 100.00 | 21.59 | 23.35 |
| UKR | Kiev | 246 | 556 | 64.23 | 59.82 | 59.35 | 47.30 | 29.25 | 31.58 |
| USA | Columbia | 452 | 203 | 77.46 | 78.71 | 100.00 | 100.00 | 19.44 | 19.98 |
| USA | New York | 210 | 275 | 74.64 | 78.39 | 100.00 | 100.00 | 21.89 | 22.89 |
| VNM | Hanoi | 455 | 255 | 76.79 | 73.81 | 100.00 | 100.00 | 18.73 | 19.49 |
| **Total** |  | 15274 | 12486 | 66.73 | 65.56 | 77.22 | 74.64 | 24.91 | 26.48 |

**Fig. S1.** Effect of COVID-19 on pro-fertility norms for each country. Dots depicts the sum of fixed and random effects from a multilevel model of pro-fertility norms reported in Table S6 (Model 2). Bars report 95% CIs of the total effect. The solid vertical line reports the fixed effect estimate of Wave 2.


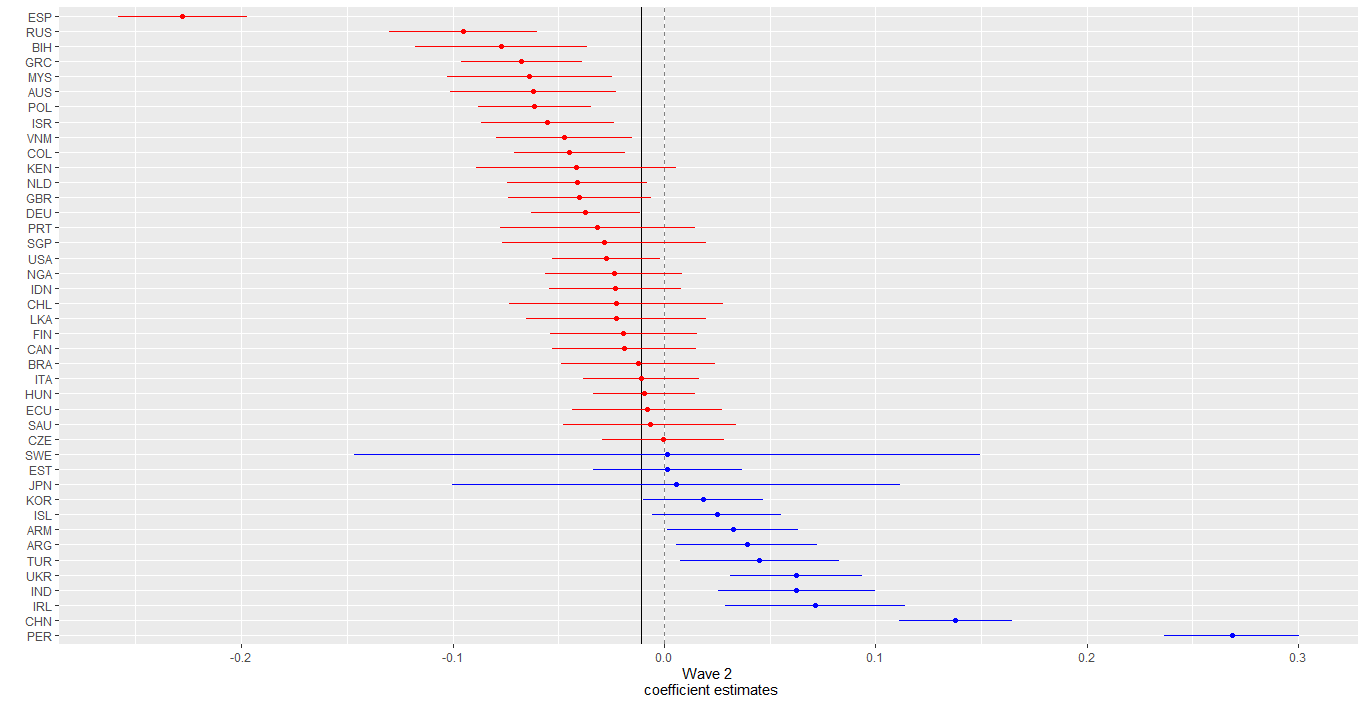


**Fig. S2.** Effect of COVID-19 on immigration threats perception for each country. Dots depicts the sum of fixed and random effects from a multilevel model of immigration threat perception reported in Table S8 (Model 2). Bars report 95% CIs of the total effect. The solid vertical line reports the fixed effect estimate of Wave 2.


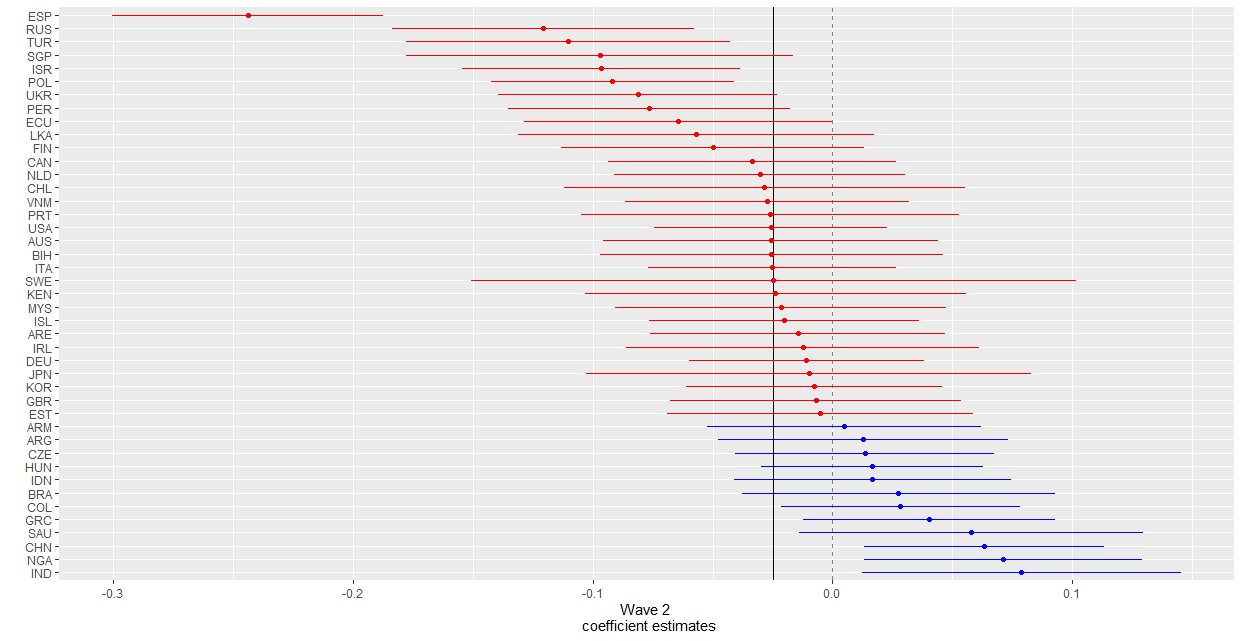


**Fig. S3.** Effect of COVID-19 on subsistence threats perception for each country. Dots depicts the sum of fixed and random effects from a multilevel model of subsistence threats perception reported in Table S9 (Model 2). Bars report 95% CIs of the total effect. The solid vertical line reports the fixed effect estimate of Wave 2.


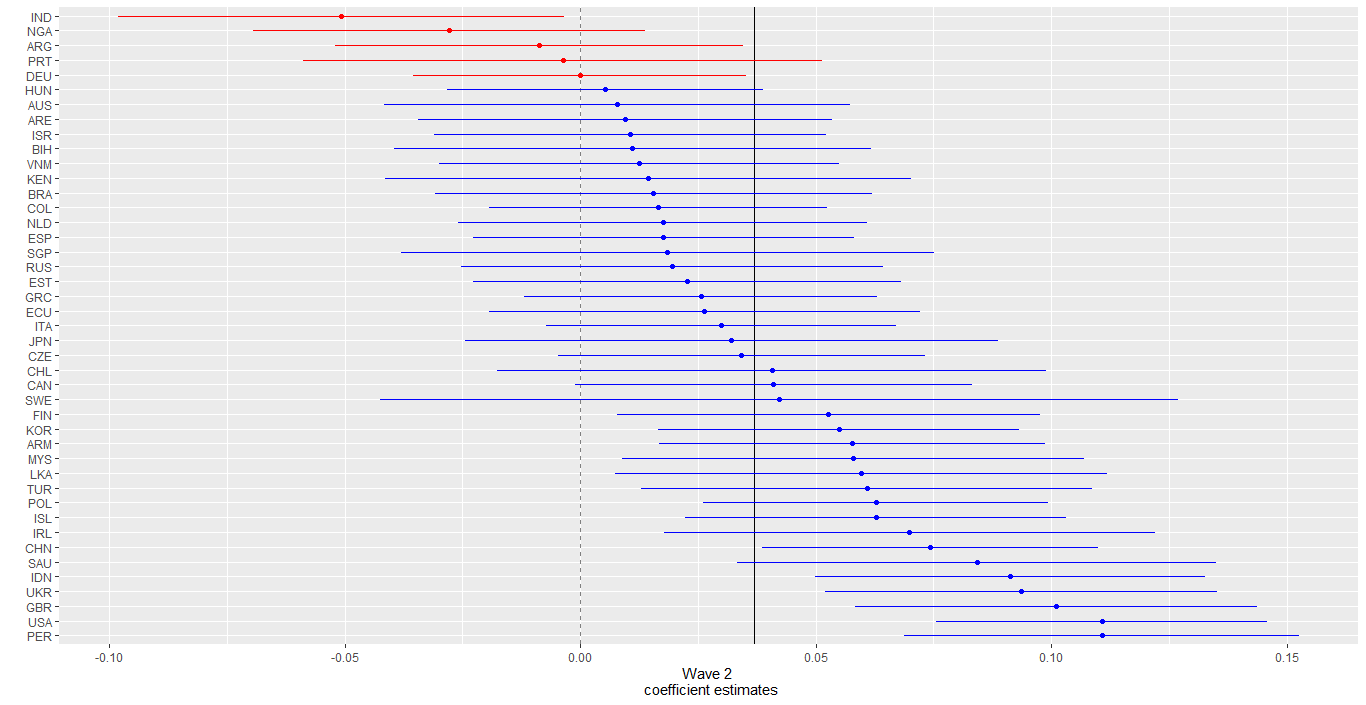


**Fig. S4.** Effect of COVID-19 on hygiene norms of spitting for each country. Dots depicts the sum of fixed and random effects from a multilevel model of hygiene norms of spitting reported in Table S12 (Model 2). Bars report 95% CIs of the total effect. The solid vertical line reports the fixed effect estimate of Wave 2.


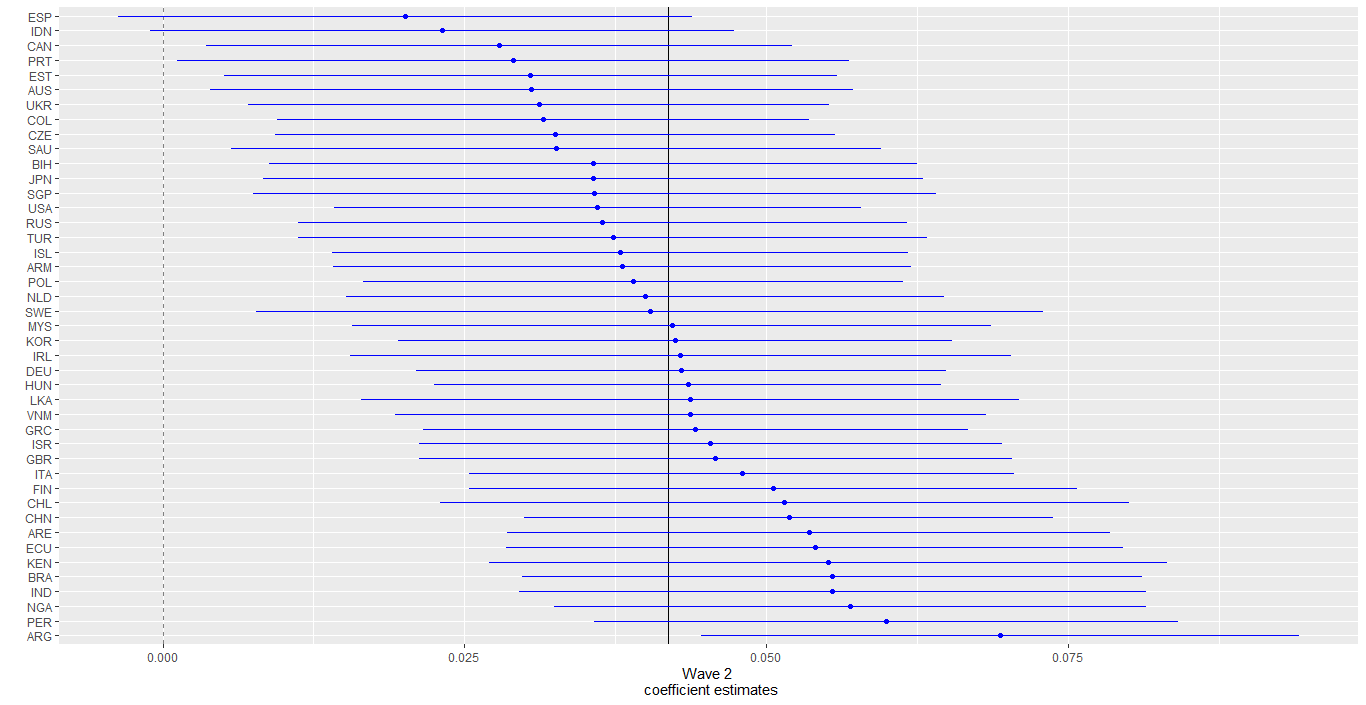


**Table S3.** OLS linear regression model with robust standard error for Collectivism. Model (1) considers observations at the country levels. (2) includes also country-level covariates to control for Covid-related variables such as number of cases and deaths (standardized). Model (3) includes interaction effect between Wave 2 and Age (to test Hypothesis 8)

|  |  |  |  |  |
| --- | --- | --- | --- | --- |
|  |  | (1) | (2) | (3) |
| *Predictors* |  | *Estimates CI p-values* | *Estimates CI p-values* | *Estimates CI p-values* |
| Wave2 |  | -0.005 -0.021 - 0.012 0.575 | -0.005 -0.021 - 0.012 0.512 | -0.042 -0.127 - 0.042 0.320 |
|  |  |  |  |  |
| Student |  | -0.048 -0.249 - 0.153 0.632 | -0.039 -0.231 - 0.153 0.684 | -0.048 -0.250 - 0.154 0.636 |
|  |  |  |  |  |
| Gender |  | -0.325 -0.615 - -0.035 **0.029** | -0.346 -0.672 - -0.020 **0.038** | -0.326 -0.617 - -0.034 **0.030** |
|  |  |  |  |  |
| Age |  | -0.003 -0.011 - 0.005 0.453 | -0.003 -0.012 - 0.005 0.472 | -0.004 -0.013 - 0.005 0.381 |
|  |  |  |  |  |
| Covid cases (sd) |  |  | 0.000 -0.026 - 0.026 0.997 |  |
|  |  |  |  |  |
| Covid death (sd) |  |  | -0.017 -0.042 - 0.008 0.186 |  |
|  |  |  |  |  |
| Wave2*Age |  |  |  | 0.001 -0.002 - 0.005 0.414 |
|  |  |  |  |  |
| Constant |  | 0.511 0.175 - 0.847 **0.004** | 0.519 0.194 - 0.844 **0.002** | 0.530 0.182 - 0.878 **0.004** |
|  |  |  |  |  |
|  |  |  |  |  |
| Observations |  | 85 | 83 | 85 |
| R-squared |  | 0.157 | 0.177 | 0.158 |

**Table S4.** OLS linear regression model with robust standard error for Duty. Model (1) considers observations at the country levels. (2) includes also country-level covariates to control for Covid-related variables such as number of cases and deaths (standardized). Model (3) includes interaction effect between Wave 2 and Age (to test Hypothesis 8)

|  |  |  |  |  |
| --- | --- | --- | --- | --- |
|  |  | (1) | (2) | (3) |
| *Predictors* |  | *Estimates CI p-values* | *Estimates CI p-values* | *Estimates CI p-values* |
| Wave2 |  | -0.000 -0.018 - 0.018 0.961 | -0.001 -0.019 - 0.017 0.918 | -0.012 -0.095 - 0.071 0.773 |
|  |  |  |  |  |
| Student |  | -0.172 -0.416 - 0.072 0.162 | -0.146 -0.356 - 0.065 0.171 | -0.172 -0.418 - 0.074 0.165 |
|  |  |  |  |  |
| Gender |  | -0.023 -0.369 - 0.323 0.895 | -0.109 -0.494 - 0.276 0.571 | -0.023 -0.371 - 0.325 0.895 |
|  |  |  |  |  |
| Age |  | -0.019 -0.028 - -0.009 **<0.001** | -0.016 -0.024 - -0.007 **0.001** | -0.019 -0.029 - -0.009 **0.001** |
|  |  |  |  |  |
| Covid cases (sd) |  |  | 0.008 -0.052 - 0.035 0.698 |  |
|  |  |  |  |  |
| Covid death (sd) |  |  | -0.017 -0.057 - 0.023 0.392 |  |
|  |  |  |  |  |
| Wave2*Age |  |  |  | 0.000 -0.003 - 0.004 0.781 |
|  |  |  |  |  |
| Constant |  | 1.094 0.635 - 1.555 **<0.001** | 1.051 0.650 - 1.453 **<0.001** | 1.100 0.637 - 1.564 **<0.001** |
|  |  |  |  |  |
|  |  |  |  |  |
| Observations |  | 85 | 83 | 85 |
| R-squared |  | 0.157 | 0.177 | 0.158 |

**Table S5.** OLS linear regression model with robust standard error for Autonomy. Model (1) considers observations at the country levels. (2) includes also country-level covariates to control for Covid-related variables such as number of cases and deaths (standardized). Model (3) includes interaction effect between Wave 2 and Age (to test Hypothesis 8)

|  |  |  |  |  |
| --- | --- | --- | --- | --- |
|  |  | (1) | (2) | (3) |
| *Predictors* |  | *Estimates CI p-values* | *Estimates CI p-values* | *Estimates CI p-values* |
| Wave2 |  | -0.002 -0.021 - 0.016 0.813 | -0.002 -0.020 - 0.016 0.839 | 0.035 -0.059 - 0.128 0.461 |
|  |  |  |  |  |
| Student |  | -0.005 -0.271 - 0.261 0.970 | -0.021 -0.244 - 0.201 0.848 | -0.005 -0.273 - 0.263 0.968 |
|  |  |  |  |  |
| Gender |  | -0.338 -0.051 - 0.727 **0.087** | 0.351 -0.088 - 0.790 0.114 | 0.338 -0.053 - 0.729 **0.089** |
|  |  |  |  |  |
| Age |  | -0.003 -0.015 - 0.008 0.542 | -0.004 -0.015 - 0.006 0.403 | -0.003 -0.014 - 0.009 **0.638** |
|  |  |  |  |  |
| Covid cases (sd) |  |  | 0.021 -0.007 - 0.050 0.139 |  |
|  |  |  |  |  |
| Covid death (sd) |  |  | -0.017 -0.057 - 0.023 0.193 |  |
|  |  |  |  |  |
| Wave2*Age |  |  |  | -0.001 -0.006 - 0.003 0.480 |
|  |  |  |  |  |
| Constant |  | 0.542 0.046 - 1.038 **0.033** | 0.565 0.143 - 0.988 **<0.010** | 0.524 0.017 - 1.031 **0.043** |
|  |  |  |  |  |
|  |  |  |  |  |
| Observations |  | 85 | 83 | 85 |
| R-squared |  | 0.157 | 0.177 | 0.158 |

| **Table S6.** Multilevel regression models of pro-fertility norms. Model (1) considers varying intercepts at the country and city level. Model (2) includes also varying slopes at the country level of the predictor Wave 2. Model (3) includes also country-level covariates to control for Covid-related variables such as number of cases and deaths (standardized). Model (4) includes interaction effect between Wave 2 and Age (to test Hypothesis 8) | | | | | | | | | | | | |
| --- | --- | --- | --- | --- | --- | --- | --- | --- | --- | --- | --- | --- |
|  | **(1)** | | | **(2)** | | | **(3)** | | | **(4)** | | |
| *Predictors* | *Estimates* | *CI* | *p-values* | *Estimates* | *CI* | *p-values* | *Estimates* | *CI* | *p-values* | *Estimates* | *CI* | *p-values* |
| Constant | 0.436 | 0.387 – 0.484 | **<0.001** | 0.431 | 0.383 – 0.479 | **<0.001** | 0.436 | 0.392 – 0.480 | **<0.001** | 0.444 | 0.395 – 0.493 | **<0.001** |
| Wave 2 | -0.010 | -0.016 – -0.005 | **<0.001** | -0.011 | -0.034 – 0.013 | 0.369 | -0.009 | -0.015 – -0.004 | **0.001** | -0.026 | -0.041 – -0.011 | **<0.001** |
| Age | 0.002 | 0.002 – 0.002 | **<0.001** | 0.002 | 0.002 – 0.002 | **<0.001** | 0.002 | 0.002 – 0.002 | **<0.001** | 0.002 | 0.001 – 0.002 | **<0.001** |
| Gender | -0.063 | -0.068 – -0.057 | **<0.001** | -0.058 | -0.064 – -0.053 | **<0.001** | -0.062 | -0.068 – -0.057 | **<0.001** | -0.063 | -0.068 – -0.058 | **<0.001** |
| Student | -0.027 | -0.035 – -0.019 | **<0.001** | -0.027 | -0.035 – -0.020 | **<0.001** | -0.027 | -0.035 – -0.019 | **<0.001** | -0.027 | -0.034 – -0.019 | **<0.001** |
| Covid Cases (sd) |  |  |  |  |  |  | -0.058 | -0.119 – 0.003 | 0.060 |  |  |  |
| Covid Deaths (sd) |  |  |  |  |  |  | -0.015 | -0.075 – 0.045 | 0.624 |  |  |  |
| Wave2*Age |  |  |  |  |  |  |  |  |  | 0.001 | 0.000 – 0.001 | **0.024** |
| **Random Effects** | | | | | | | | | | | | |
| σ^2^ | 0.04 | | | 0.04 | | | 0.05 | | | 0.04 | | |
| τ_00_ | 0.00 _City_code_ | | | 0.00 _City_code_ | | | 0.00 _City_code_ | | | 0.00 _City_code_ | | |
|  | 0.02 _Country_ISO_Code_ | | | 0.02 _Country_ISO_Code_ | | | 0.02 _Country_ISO_Code_ | | | 0.02 _Country_ISO_Code_ | | |
| τ_11_ |  | | | 0.01 _Country_ISO_Code.Wave2_ | | |  | | |  | | |
| ρ_01_ |  | | | -0.12 _Country_ISO_Code_ | | |  | | |  | | |
| ICC | 0.35 | | | 0.36 | | | 0.29 | | | 0.35 | | |
| N | 42 _Country_ISO_Code_ | | | 42 _Country_ISO_Code_ | | | 41 _Country_ISO_Code_ | | | 42 _Country_ISO_Code_ | | |
|  | 54 _City_code_ | | | 54 _City_code_ | | | 53 _City_code_ | | | 54 _City_code_ | | |
| Observations | 28623 | | | 28623 | | | 27929 | | | 28623 | | |
| Marginal R^2^ / Conditional R^2^ | 0.024 / 0.364 | | | 0.023 / 0.378 | | | 0.093 / 0.360 | | | 0.024 / 0.364 | | |
| **Table S7.** Multilevel regression models of immigration threats perception. Model (1) considers varying intercepts at the country and city level. Model (2) includes also varying slopes at the country level of the predictor Wave 2. Model (3) includes also country-level covariates to control for Covid-related variables such as number of cases and deaths (standardized). Model (4) includes interaction effect between Wave 2 and Age (to test Hypothesis 8). | | | | | | | | | | | | |
|  | **(1)** | | | **(2)** | | | **(3)** | | | **(4)** | | |
| *Predictors* | *Estimates* | *CI* | *p-values* | *Estimates* | *CI* | *p-values* | *Estimates* | *CI* | *p-values* | *Estimates* | *CI* | *p-values* |
| Constant | 0.299 | 0.242 – 0.356 | **<0.001** | 0.303 | 0.244 – 0.362 | **<0.001** | 0.302 | 0.244 – 0.360 | **<0.001** | 0.294 | 0.235 – 0.352 | **<0.001** |
| Wave 2 | -0.022 | -0.033 – -0.012 | **<0.001** | -0.025 | -0.048 – -0.002 | **0.035** | -0.022 | -0.033 – -0.012 | **<0.001** | -0.013 | -0.041 – 0.016 | 0.391 |
| Age | 0.001 | 0.000 – 0.002 | **0.013** | 0.001 | 0.000 – 0.001 | **0.027** | 0.001 | 0.000 – 0.002 | **0.013** | 0.001 | 0.000 – 0.002 | **0.019** |
| Gender | -0.013 | -0.024 – -0.003 | **0.015** | -0.012 | -0.023 – -0.001 | **0.026** | -0.013 | -0.024 – -0.003 | **0.015** | -0.013 | -0.024 – -0.002 | **0.016** |
| Student | -0.010 | -0.025 – 0.006 | 0.221 | -0.011 | -0.026 – 0.005 | 0.186 | -0.009 | -0.025 – 0.006 | 0.232 | -0.010 | -0.025 – 0.006 | 0.215 |
| Covid Cases (sd) |  |  |  |  |  |  | 0.009 | -0.067 – 0.084 | 0.823 |  |  |  |
| Covid Deaths (sd) |  |  |  |  |  |  | -0.034 | -0.108 – 0.040 | 0.369 |  |  |  |
| Wave2*Age |  |  |  |  |  |  |  |  |  | -0.000 | -0.001 – 0.001 | 0.479 |
| **Random Effects** | | | | | | | | | | | | |
| σ^2^ | 0.18 | | | 0.18 | | | 0.18 | | | 0.18 | | |
| τ_00_ | 0.00 _City_code_ | | | 0.00 _City_code_ | | | 0.00 _City_code_ | | | 0.00 _City_code_ | | |
|  | 0.02 _Country_ISO_Code_ | | | 0.03 _Country_ISO_Code_ | | | 0.03 _Country_ISO_Code_ | | | 0.02 _Country_ISO_Code_ | | |
| τ_11_ |  | | | 0.00 _Country_ISO_Code.Wave2_ | | |  | | |  | | |
| ρ_01_ |  | | | -0.34 _Country_ISO_Code_ | | |  | | |  | | |
| ICC | 0.14 | | | 0.14 | | | 0.14 | | | 0.14 | | |
| N | 43 _Country_ISO_Code_ | | | 43 _Country_ISO_Code_ | | | 42 _Country_ISO_Code_ | | | 43 _Country_ISO_Code_ | | |
|  | 55 _City_code_ | | | 55 _City_code_ | | | 54 _City_code_ | | | 55 _City_code_ | | |
| Observations | 29434 | | | 29434 | | | 28734 | | | 29434 | | |
| Marginal R^2^ / Conditional R^2^ | 0.001 / 0.136 | | | 0.001 / 0.139 | | | 0.005 / 0.142 | | | 0.001 / 0.136 | | |
| **Table S8.** Multilevel regression models of subsistence threats perception. Model (1) considers varying intercepts at the country and city level. Model (2) includes also varying slopes at the country level of the predictor Wave 2. Model (3) includes also country-level covariates to control for Covid-related variables such as number of cases and deaths (standardized). Model (4) includes interaction effect between Wave 2 and Age (to test Hypothesis 8) | | | | | | | | | | | | |
|  | **(1)** | | | **(2)** | | | **(3)** | | | **(4)** | | |
| *Predictors* | *Estimates* | *CI* | *p-values* | *Estimates* | *CI* | *p-values* | *Estimates* | *CI* | *p-values* | *Estimates* | *CI* | *p-values* |
| Constant | 0.442 | 0.394 – 0.491 | **<0.001** | 0.438 | 0.388 – 0.487 | **<0.001** | 0.441 | 0.392 – 0.490 | **<0.001** | 0.457 | 0.408 – 0.507 | **<0.001** |
| Wave 2 | 0.039 | 0.031 – 0.046 | **<0.001** | 0.037 | 0.022 – 0.052 | **<0.001** | 0.039 | 0.032 – 0.047 | **<0.001** | 0.010 | -0.011 – 0.031 | 0.346 |
| Age | -0.001 | -0.001 – -0.000 | **0.029** | -0.000 | -0.001 – 0.000 | 0.121 | -0.001 | -0.001 – -0.000 | **0.033** | -0.001 | -0.002 – -0.001 | **<0.001** |
| Gender | 0.061 | 0.053 – 0.068 | **<0.001** | 0.062 | 0.054 – 0.069 | **<0.001** | 0.059 | 0.051 – 0.067 | **<0.001** | 0.060 | 0.053 – 0.068 | **<0.001** |
| Student | 0.014 | 0.002 – 0.025 | **0.018** | 0.015 | 0.003 – 0.026 | **0.012** | 0.014 | 0.003 – 0.025 | **0.016** | 0.014 | 0.003 – 0.026 | **0.015** |
| Covid Cases (sd) |  |  |  |  |  |  | -0.038 | -0.104 – 0.027 | 0.254 |  |  |  |
| Covid Deaths (sd) |  |  |  |  |  |  | 0.015 | -0.050 – 0.079 | 0.655 |  |  |  |
| Wave2*Age |  |  |  |  |  |  |  |  |  | 0.001 | 0.000 – 0.002 | **0.004** |
| **Random Effects** | | | | | | | | | | | | |
| σ^2^ | 0.10 | | | 0.10 | | | 0.10 | | | 0.10 | | |
| τ_00_ | 0.00 _City_code_ | | | 0.00 _City_code_ | | | 0.00 _City_code_ | | | 0.00 _City_code_ | | |
|  | 0.02 _Country_ISO_Code_ | | | 0.02 _Country_ISO_Code_ | | | 0.02 _Country_ISO_Code_ | | | 0.02 _Country_ISO_Code_ | | |
| τ_11_ |  | | | 0.00 _Country_ISO_Code.Wave2_ | | |  | | |  | | |
| ρ_01_ |  | | | -0.15 _Country_ISO_Code_ | | |  | | |  | | |
| ICC | 0.18 | | | 0.19 | | | 0.18 | | | 0.18 | | |
| N | 43 _Country_ISO_Code_ | | | 43 _Country_ISO_Code_ | | | 42 _Country_ISO_Code_ | | | 43 _Country_ISO_Code_ | | |
|  | 55 _City_code_ | | | 55 _City_code_ | | | 54 _City_code_ | | | 55 _City_code_ | | |
| Observations | 29434 | | | 29434 | | | 28734 | | | 29434 | | |
| Marginal R^2^ / Conditional R^2^ | 0.011 / 0.193 | | | 0.010 / 0.197 | | | 0.018 / 0.198 | | | 0.011 / 0.193 | | |
| **Table S9.** Multilevel regression models of hygiene norms of spitting. Model (1) considers varying intercepts at the country and city level. Model (2) includes also varying slopes at the country level of the predictor Wave 2. Model (3) includes also country-level covariates to control for Covid-related variables such as number of cases and deaths (standardized). Model (4) includes interaction effect between Wave 2 and Age (to test Hypothesis 8) | | | | | | | | | | | | |
|  | **(1)** | | | **(2)** | | | **(3)** | | | **(4)** | | |
| *Predictors* | *Estimates* | *CI* | *p-values* | *Estimates* | *CI* | *p-values* | *Estimates* | *CI* | *p-values* | *Estimates* | *CI* | *p-values* |
| Constant | 0.510 | 0.475 – 0.545 | **<0.001** | 0.512 | 0.477 – 0.547 | **<0.001** | 0.512 | 0.475 – 0.548 | **<0.001** | 0.520 | 0.484 – 0.555 | **<0.001** |
| Wave 2 | 0.042 | 0.036 – 0.047 | **<0.001** | 0.042 | 0.034 – 0.050 | **<0.001** | 0.042 | 0.036 – 0.047 | **<0.001** | 0.024 | 0.008 – 0.040 | **0.004** |
| Age | 0.002 | 0.001 – 0.002 | **<0.001** | 0.002 | 0.001 – 0.002 | **<0.001** | 0.002 | 0.001 – 0.002 | **<0.001** | 0.001 | 0.001 – 0.002 | **<0.001** |
| Gender | 0.090 | 0.084 – 0.096 | **<0.001** | 0.090 | 0.084 – 0.096 | **<0.001** | 0.090 | 0.084 – 0.096 | **<0.001** | 0.090 | 0.084 – 0.096 | **<0.001** |
| Student | 0.002 | -0.007 – 0.010 | 0.711 | 0.001 | -0.008 – 0.010 | 0.811 | 0.002 | -0.007 – 0.010 | 0.729 | 0.002 | -0.007 – 0.011 | 0.673 |
| Covid Cases (sd) |  |  |  |  |  |  | -0.012 | -0.061 – 0.037 | 0.632 |  |  |  |
| Covid Deaths (sd) |  |  |  |  |  |  | 0.006 | -0.042 – 0.054 | 0.801 |  |  |  |
| Wave2*Age |  |  |  |  |  |  |  |  |  | 0.001 | 0.000 – 0.001 | **0.018** |
| **Random Effects** | | | | | | | | | | | | |
| σ^2^ | 0.06 | | | 0.06 | | | 0.06 | | | 0.06 | | |
| τ_00_ | 0.00 _City_code_ | | | 0.00 _City_code_ | | | 0.00 _City_code_ | | | 0.00 _City_code_ | | |
|  | 0.01 _Country_ISO_Code_ | | | 0.01 _Country_ISO_Code_ | | | 0.01 _Country_ISO_Code_ | | | 0.01 _Country_ISO_Code_ | | |
| τ_11_ |  | | | 0.00 _Country_ISO_Code.Wave2_ | | |  | | |  | | |
| ρ_01_ |  | | | 0.08 _Country_ISO_Code_ | | |  | | |  | | |
| ICC | 0.17 | | | 0.17 | | | 0.18 | | | 0.17 | | |
| N | 43 _Country_ISO_Code_ | | | 43 _Country_ISO_Code_ | | | 42 _Country_ISO_Code_ | | | 43 _Country_ISO_Code_ | | |
|  | 55 _City_code_ | | | 55 _City_code_ | | | 54 _City_code_ | | | 55 _City_code_ | | |
| Observations | 29032 | | | 29032 | | | 28336 | | | 29032 | | |
| Marginal R^2^ / Conditional R^2^ | 0.036 / 0.197 | | | 0.036 / 0.198 | | | 0.037 / 0.208 | | | 0.036 / 0.197 | | |

**Table S10.** OLS models of change in Pro-fertility norms. Observations at the country level. Heteroskedasticity-robust errors.

|  |  | (1) |
| --- | --- | --- |
| *Predictors* |  | *Estimates CI p-values* |
| Student |  | -0.072 -0.233 - 0.090 0.373 |
|  |  |  |
| Gender |  | 0.139 -0.110 - 0.389 0.264 |
|  |  |  |
| Age |  | 0.002 -0.007 - 0.011 0.701 |
|  |  |  |
| Fear |  | 0.083 -0.145 - 0.311 0.463 |
|  |  |  |
| Perc. Prev. |  | -0.155 -0.622 - 0.312 0.505 |
|  |  |  |
| Stringency |  | -0.009 -0.025 - 0.006 0.238 |
|  |  |  |
| Constant |  | -0.107 -0.487 - 0.272 0.569 |
|  |  |  |
|  |  |  |
| Observations |  | 40 |
| R-squared |  | 0.256 |

**Table S11.** OLS models of change in Immigration threat perception. Observations at the country level. Heteroskedasticity-robust errors.

|  |  | (1) |
| --- | --- | --- |
| *Predictors* |  | *Estimates CI p-values* |
| Student |  | -0.095 -0.230 - 0.040 0.162 |
|  |  |  |
| Gender |  | 0.160 -0.112 - 0.432 0.241 |
|  |  |  |
| Age |  | -0.004 -0.012 - 0.004 0.361 |
|  |  |  |
| Fear |  | 0.089 -0.223 - 0.401 0.565 |
|  |  |  |
| Perc. Prev. |  | -0.359 -0.886 - 0.167 0.174 |
|  |  |  |
| Stringency |  | -0.012 -0.029 - 0.005 0.169 |
|  |  |  |
| Constant |  | 0.060 -0.360 - 0.480 0.773 |
|  |  |  |
|  |  |  |
| Observations |  | 40 |
| R-squared |  | 0.179 |

**Table S12.** OLS models of change in Subsistence threat perception. Observations at the country level. Heteroskedasticity-robust errors.

|  |  | (1) |
| --- | --- | --- |
| *Predictors* |  | *Estimates CI p-values* |
| Student |  | -0.079 -0.207 - 0.050 0.223 |
|  |  |  |
| Gender |  | 0.052 -0.093 - 0.197 0.470 |
|  |  |  |
| Age |  | -0.002 -0.008 - 0.003 0.413 |
|  |  |  |
| Fear |  | 0.130 -0.080 - 0.340 0.217 |
|  |  |  |
| Perc. Prev. |  | -0.079 -0.442 - 0.285 0.663 |
|  |  |  |
| Stringency |  | -0.012 -0.028 - 0.005 0.175 |
|  |  |  |
| Constant |  | 0.051 -0.209 - 0.312 0.691 |
|  |  |  |
|  |  |  |
| Observations |  | 40 |
| R-squared |  | 0.113 |

**Table S13.** OLS models of change in hygiene norms of Spitting. Observations at the country level. Heteroskedasticity-robust errors.

|  |  | (1) |
| --- | --- | --- |
| *Predictors* |  | *Estimates CI p-values* |
| Student |  | 0.040 -0.032 - 0.111 0.269 |
|  |  |  |
| Gender |  | -0.011 -0.110 - 0.088 0.821 |
|  |  |  |
| Age |  | 0.004 0.002 - 0.007 0.004 |
|  |  |  |
| Fear |  | 0.148 0.035 - 0.262 **0.012** |
|  |  |  |
| Perc. Prev. |  | -0.054 -0.212 - 0.105 0.497 |
|  |  |  |
| Stringency |  | 0.011 0.003 - 0.019 **0.007** |
|  |  |  |
| Constant |  | -0.183 -0.340 - -0.027 **0.023** |
|  |  |  |
|  |  |  |
| Observations |  | 40 |
| R-squared |  | 0.404 |

**Table S14.** Comparison of original analyses (OLS or MLM) and OLS fixed-effects analyses with clustered robust standard errors (OLS FE).

|  | **Collectivism** | | **Duty** | | **Autonomy** | | **Fertility** | | **Immigration** | | **Subsistence** | | **Spitting** | |
| --- | --- | --- | --- | --- | --- | --- | --- | --- | --- | --- | --- | --- | --- | --- |
|  | *OLS* | *OLS FE* | *OLS* | *OLS FE* | *OLS* | *OLS FE* | *MLM* | *OLS FE* | *MLM* | *OLS FE* | *MLM* | *OLS FE* | *MLM* | *OLS FE* |
| Wave 2 | -0.005 | -0.007 | -0.000 | -0.001 | -0.002 | -0.000 | -0.010*** | -0.006 | -0.022*** | -0.021+ | 0.039*** | 0.039*** | 0.042*** | 0.041*** |
|  | (-0.021; 0.012) | (-0.028; 0.014) | (-0.018; 0.018) | (-0.026; 0.024) | (-0.021; 0.016) | (-0.025; 0.025) | (-0.016;  -0.005) | (-0.032; 0.019) | (-0.033;  -0.012) | (-0.046; 0.003) | (0.031;  0.046) | (0.023; 0.055) | (0.036; 0.047) | (0.033; 0.049) |
| Observations | 85 | 85 | 85 | 85 | 85 | 85 | 28263 | 28263 | 29434 | 29434 | 29434 | 29434 | 29032 | 29032 |

*Notes*: OLS or MLM models refer to original analyses. OLS FE models are the additional analyses using country-level fixed effects with cluster robust standard errors at the country level. The coefficient “Wave 2” indicates the change in outcome between the two waves. Standard errors in parentheses. All models include controls for age, gender, and student status.

^+^ *p* < 0.10, ^*^ *p* < 0.05, ^**^ *p* < 0.01, ^***^ *p* < 0.001

**Table S15** Variation in outcomes across Wave 1 and Wave 2.

|  | **Collectivism** | | **Duty** | | **Autonomy** | | **Fertility** | | **Immigration** | | **Subsistence** | | **Spitting** | |
| --- | --- | --- | --- | --- | --- | --- | --- | --- | --- | --- | --- | --- | --- | --- |
|  | *Wave 1* | *Wave 2* | *Wave 1* | *Wave 2* | *Wave 1* | *Wave 2* | *Wave 1* | *Wave 2* | *Wave 1* | *Wave 2* | *Wave 1* | *Wave 2* | *Wave 1* | *Wave 2* |
| SD | 0.10 | 0.10 | 0.15 | 0.14 | 0.11 | 0.11 | 0.26 | 0.27 | 0.45 | 0.45 | 0.35 | 0.33 | 0.26 | 0.27 |
| Observations | 43 | 42 | 43 | 42 | 43 | 42 | 14882 | 14059 | 15274 | 14486 | 15274 | 14486 | 15108 | 14244 |

**Figure S5.** Variation in outcomes across Wave 1 and Wave 2.


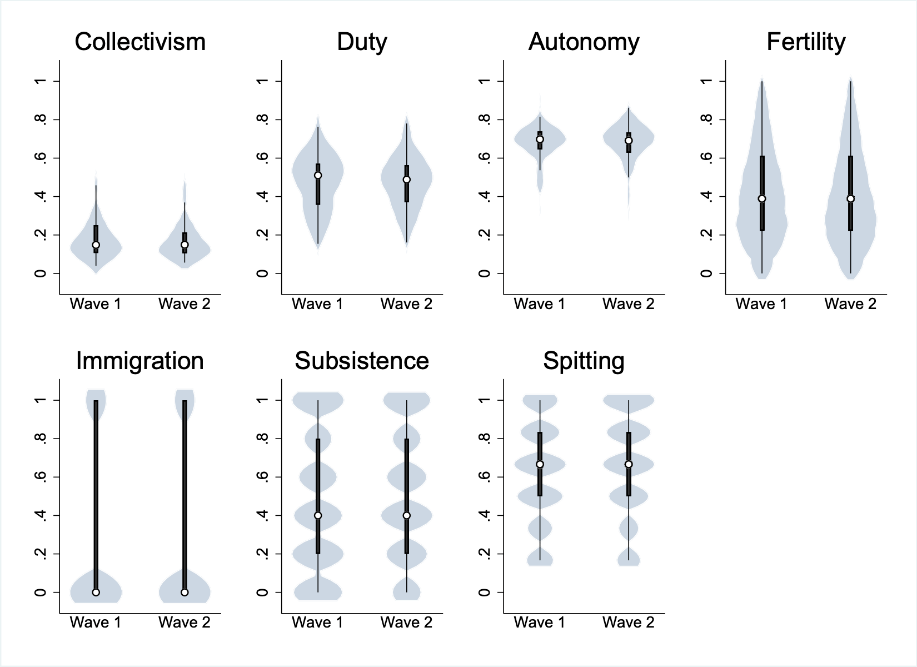


**Table S16.** Concepts, dimensions, wording and interpretation of all items not used and studied in the main analysis.

| **Concept** | **Dimensions** | **Wording** | **Interpretation** |
| --- | --- | --- | --- |
| *Hofstede’s cultural dimension*^[4]^ | Individualism/  collectivism | Please think of an ideal job, disregarding your present job, if you have one. In choosing an ideal job, how important would it be to you to...   - … have sufficient time for your personal or home life? - … have security of employment? - … do work that is interesting? - … have a job respected by your family and friends? | Low values: individualism  High values: collectivism |
|  | Indulgence/  restraint | - In your private life, how important is keeping time free for fun? - In your private life, how important is moderation: having few desires? - Are you a happy person? - Do other people or circumstances ever prevent you from doing what you really want to? | Low values: indulgence (satisfaction is good)  High values: restraint (normative repression) |
|  | Power distance | Please think of an ideal job, disregarding your present job, if you have one. In choosing an ideal job, how important would it be to you to...   - … have a boss (direct superior) you can respect? - … be consulted by your boss in decisions involving your work? - How often, in your experience, are subordinates afraid to contradict their boss (or students their teacher)? - To what extent do you agree or disagree with: an organization structure in which certain subordinates have two bosses should be avoided at all cost | Low values: egalitarian  High values: embraces hierarchy |
|  |  | Which of the following do you think of as real threats to your society (tick all  that apply) |  |
| *Perceived societal threats*^[3]^ | Demographic threat | - Overpopulation | Low values: less perception as real threat.  High values: more perception as real threat. |
|  |  |  |  |
|  | Conflict threat | - Conflict within the countries - Conflict with other countries |  |
| *Justification of violence* (World Values Survey) |  | Please tell us for each of the following actions whether you think it can always be justified, never be justified, or something in between   - For a man to beat his wife - To use violence against other people | Low values: less violence  High values: more violence |
| *Hygiene norm*^[3]^ | Brushing teeth regularly | - How often do you think people should brush their teeth? | Low values: low hygiene  High values: high hygiene |

**Fig. S6.** Effect of COVID-19 on conflict threats perception for each country. Dots depicts the sum of fixed and random effects from a multilevel model of conflict threats perception reported in Table S10 (Model 2). Bars report 95% CIs of the total effect. The solid vertical line reports the fixed effect estimate of Wave 2.


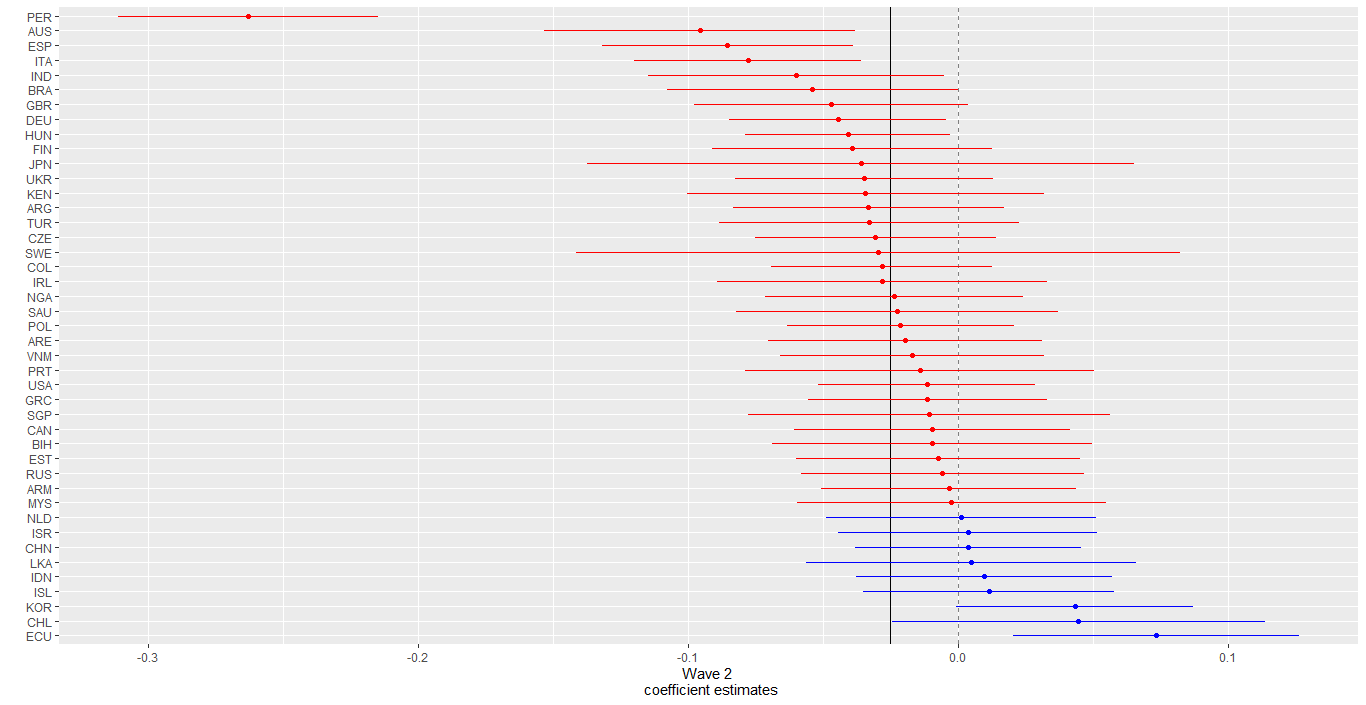


**Fig. S7.** Effect of COVID-19 on justification of violence for each country. Dots depicts the sum of fixed and random effects from a multilevel model of justification of violence reported in Table S11 (Model 2). Bars report 95% CIs of the total effect. The solid vertical line reports the fixed effect estimate of Wave 2.


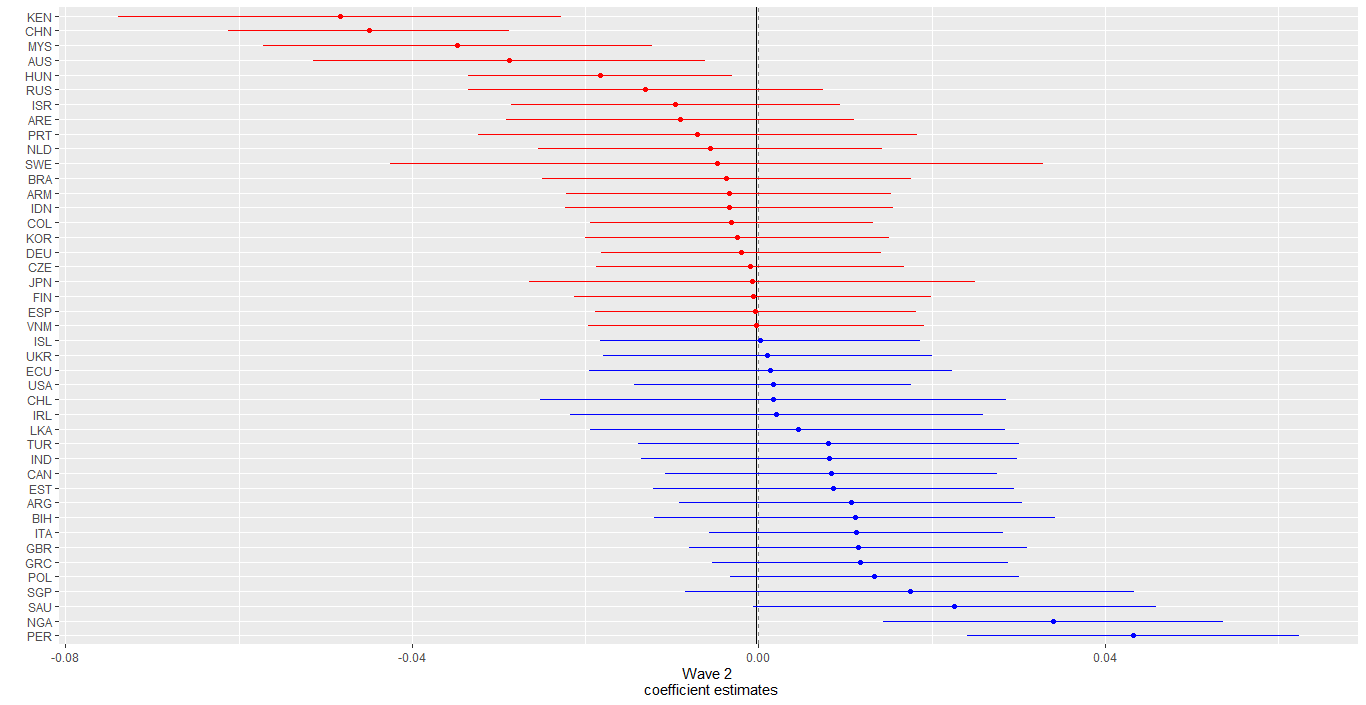


**Fig. S8.** Effect of COVID-19 on hygiene norms of brushing teeth for each country. Dots depicts the sum of fixed and random effects from a multilevel model of hygiene norms of brushing teeth reported in Table S13 (Model 2). Bars report 95% CIs of the total effect. The solid vertical line reports the fixed effect estimate of Wave 2.


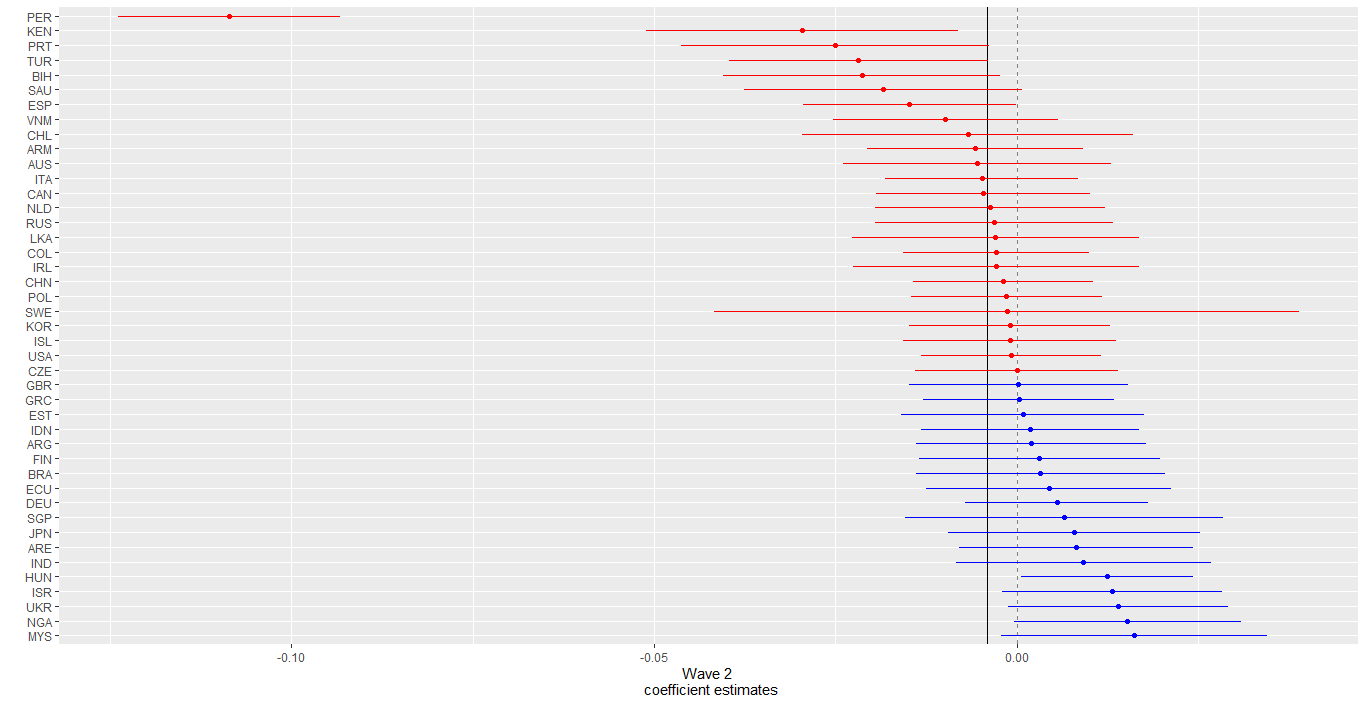


| **Table S17.** OLS linear regression model with robust standard error for Hofstede’s dimension of collectivism. Model (1) considers observations at the country levels. (2) includes also country-level covariates to control for Covid-related variables such as number of cases and deaths (standardized). Model (3) includes interaction effect between Wave 2 and Age (to test Hypothesis 8) | | | | | | | | | |
| --- | --- | --- | --- | --- | --- | --- | --- | --- | --- |
|  | **(1)** | | | **(2)** | | | **(3)** | | |
| *Predictors* | *Estimates* | *CI* | *p* | *Estimates* | *CI* | *p* | *Estimates* | *CI* | *p* |
| Constant | 0.569 | 0.465 – 0.673 | **<0.001** | 0.565 | 0.479 – 0.651 | **<0.001** | 0.576 | 0.462 – 0.690 | **<0.001** |
| Wave 2 | 0.008 | -0.005 – 0.021 | **0.003** | 0.007 | -0.004 – 0.019 | 0.210 | -0.006 | -0.085 – 0.074 | 0.890 |
| Age | -0.002 | -0.005 – 0.000 | 0.060 | -0.001 | -0.004 – 0.001 | 0.174 | -0.003 | -0.006 – 0.000 | 0.080 |
| Gender | -0.096 | -0.161 – -0.031 | **0.004** | -0.140 | -0.207 – -0.074 | **<0.001** | -0.096 | -0.161 – -0.030 | **0.005** |
| Student | -0.006 | -0.061 – 0.050 | 0.833 | 0.003 | -0.042 – 0.049 | 0.884 | -0.006 | -0.062 – 0.050 | 0.837 |
| Covid Cases (sd) |  |  |  | 0.007 | 0.000 – 0.013 | **0.036** |  |  |  |
| Covid Deaths (sd) |  |  |  | -0.016 | -0.022 – -0.009 | **<0.001** |  |  |  |
| Wave2*Age |  |  |  |  |  |  | 0.001 | -0.002 – 0.004 | 0.725 |
| Observations | 85 | | | 83 | | | 85 | | |
| R^2^ / R^2^ adjusted | 0.254 / 0.216 | | | 0.389 / 0.341 | | | 0.255 / 0.208 | | |

| **Table S18.** OLS linear regression model with robust standard error for Hofstede’s dimension of restraint. Model (1) considers observations at the country levels. (2) includes also country-level covariates to control for Covid-related variables such as number of cases and deaths (standardized). Model (3) includes interaction effect between Wave 2 and Age (to test Hypothesis 8) | | | | | | | | | |
| --- | --- | --- | --- | --- | --- | --- | --- | --- | --- |
|  | **(1)** | | | **(2)** | | | **(3)** | | |
| *Predictors* | *Estimates* | *CI* | *p* | *Estimates* | *CI* | *p* | *Estimates* | *CI* | *p* |
| Constant | 0.508 | 0.410 – 0.607 | **<0.001** | 0.492 | 0.402 – 0.582 | **<0.001** | 0.524 | 0.408 – 0.639 | **<0.001** |
| Wave 2 | 0.006 | -0.010 – 0.023 | **0.095** | 0.006 | -0.009 – 0.022 | 0.420 | -0.025 | -0.125 – 0.075 | 0.623 |
| Age | -0.003 | -0.005 – -0.000 | **0.019** | -0.002 | -0.004 – 0.000 | 0.106 | -0.003 | -0.007 – -0.000 | **0.042** |
| Gender | -0.019 | -0.118 – 0.080 | 0.701 | -0.050 | -0.158 – 0.058 | 0.355 | -0.019 | -0.119 – 0.080 | 0.701 |
| Student | -0.037 | -0.086 – 0.012 | 0.134 | -0.029 | -0.074 – 0.017 | 0.214 | -0.037 | -0.087 – 0.012 | 0.140 |
| Covid Cases (sd) |  |  |  | -0.002 | -0.010 – 0.006 | 0.599 |  |  |  |
| Covid Deaths (sd) |  |  |  | -0.005 | -0.015 – 0.004 | 0.256 |  |  |  |
| Wave2*Age |  |  |  |  |  |  | 0.001 | -0.003 – 0.005 | 0.526 |
| Observations | 85 | | | 83 | | | 85 | | |
| R^2^ / R^2^ adjusted | 0.070 / 0.023 | | | 0.104 / 0.033 | | | 0.076 / 0.017 | | |

| **Table S19.** OLS linear regression model with robust standard error for Hofstede’s dimension of power distance. Model (1) considers observations at the country levels. (2) includes also country-level covariates to control for Covid-related variables such as number of cases and deaths (standardized). Model (3) includes interaction effect between Wave 2 and Age (to test Hypothesis 8) | | | | | | | | | |
| --- | --- | --- | --- | --- | --- | --- | --- | --- | --- |
|  | **(1)** | | | **(2)** | | | **(3)** | | |
| *Predictors* | *Estimates* | *CI* | *p* | *Estimates* | *CI* | *p* | *Estimates* | *CI* | *p* |
| Constant | 0.635 | 0.542 – 0.728 | **<0.001** | 0.636 | 0.542 – 0.731 | **<0.001** | 0.640 | 0.537 – 0.743 | **<0.001** |
| Wave 2 | 0.003 | -0.008 – 0.014 | 0.192 | 0.003 | -0.008 – 0.013 | 0.640 | -0.007 | -0.071 – 0.057 | 0.833 |
| Age | -0.002 | -0.004 – 0.001 | 0.144 | -0.001 | -0.004 – 0.001 | 0.247 | -0.002 | -0.005 – 0.001 | 0.185 |
| Gender | -0.023 | -0.073 – 0.027 | 0.370 | -0.042 | -0.096 – 0.013 | 0.133 | -0.023 | -0.074 – 0.028 | 0.376 |
| Student | -0.035 | -0.076 – 0.006 | 0.093 | -0.032 | -0.073 – 0.008 | 0.117 | -0.035 | -0.076 – 0.007 | 0.100 |
| Covid Cases (sd) |  |  |  | 0.006 | -0.002 – 0.013 | 0.138 |  |  |  |
| Covid Deaths (sd) |  |  |  | -0.007 | -0.014 – 0.000 | 0.061 |  |  |  |
| Wave2*Age |  |  |  |  |  |  | 0.000 | -0.002 – 0.003 | 0.763 |
| Observations | 85 | | | 83 | | | 85 | | |
| R^2^ / R^2^ adjusted | 0.074 / 0.028 | | | 0.112 / 0.042 | | | 0.075 / 0.017 | | |

| **Table S20.** Multilevel regression models of conflict threats perception. Model (1) considers varying intercepts at the country and city level. Model (2) includes also varying slopes at the country level of the predictor Wave 2. Model (3) includes also country-level covariates to control for Covid-related variables such as number of cases and deaths (standardized). Model (4) includes interaction effect between Wave 2 and Age (to test Hypothesis 8) | | | | | | | | | | | | |
| --- | --- | --- | --- | --- | --- | --- | --- | --- | --- | --- | --- | --- |
|  | **(1)** | | | **(2)** | | | **(3)** | | | **(4)** | | |
| *Predictors* | *Estimates* | *CI* | *p* | *Estimates* | *CI* | *p* | *Estimates* | *CI* | *p* | *Estimates* | *CI* | *p* |
| Constant | 0.633 | 0.583 – 0.683 | **<0.001** | 0.635 | 0.586 – 0.685 | **<0.001** | 0.636 | 0.585 – 0.687 | **<0.001** | 0.640 | 0.589 – 0.692 | **<0.001** |
| Wave 2 | -0.027 | -0.035 – -0.018 | **<0.001** | -0.025 | -0.044 – -0.005 | **0.012** | -0.027 | -0.035 – -0.018 | **<0.001** | -0.041 | -0.065 – -0.018 | **0.001** |
| Age | -0.002 | -0.002 – -0.001 | **<0.001** | -0.002 | -0.003 – -0.001 | **<0.001** | -0.002 | -0.002 – -0.001 | **<0.001** | -0.002 | -0.003 – -0.002 | **<0.001** |
| Gender | 0.036 | 0.028 – 0.045 | **<0.001** | 0.035 | 0.027 – 0.044 | **<0.001** | 0.036 | 0.027 – 0.045 | **<0.001** | 0.036 | 0.028 – 0.045 | **<0.001** |
| Student | 0.022 | 0.009 – 0.035 | **0.001** | 0.021 | 0.008 – 0.034 | **0.001** | 0.022 | 0.009 – 0.035 | **0.001** | 0.022 | 0.009 – 0.035 | **0.001** |
| Covid Cases (sd) |  |  |  |  |  |  | -0.041 | -0.108 – 0.026 | 0.231 |  |  |  |
| Covid Deaths (sd) |  |  |  |  |  |  | 0.009 | -0.057 – 0.076 | 0.781 |  |  |  |
| Wave2*Age |  |  |  |  |  |  |  |  |  | 0.001 | -0.000 – 0.001 | 0.194 |
| **Random Effects** | | | | | | | | | | | | |
| σ^2^ | 0.12 | | | 0.12 | | | 0.12 | | | 0.12 | | |
| τ_00_ | 0.00 _City_code_ | | | 0.00 _City_code_ | | | 0.00 _City_code_ | | | 0.00 _City_code_ | | |
|  | 0.02 _Country_ISO_Code_ | | | 0.02 _Country_ISO_Code_ | | | 0.02 _Country_ISO_Code_ | | | 0.02 _Country_ISO_Code_ | | |
| τ_11_ |  | | | 0.00 _Country_ISO_Code.Wave2_ | | |  | | |  | | |
| ρ_01_ |  | | | 0.02 _Country_ISO_Code_ | | |  | | |  | | |
| ICC | 0.16 | | | 0.16 | | | 0.16 | | | 0.16 | | |
| N | 43 _Country_ISO_Code_ | | | 43 _Country_ISO_Code_ | | | 42 _Country_ISO_Code_ | | | 43 _Country_ISO_Code_ | | |
|  | 55 _City_code_ | | | 55 _City_code_ | | | 54 _City_code_ | | | 55 _City_code_ | | |
| Observations | 29434 | | | 29434 | | | 28734 | | | 29434 | | |
| Marginal R^2^ / Conditional R^2^ | 0.008 / 0.163 | | | 0.008 / 0.167 | | | 0.016 / 0.173 | | | 0.008 / 0.163 | | |
| **Table S21.** Multilevel regression models of justification of violence. Model (1) considers varying intercepts at the country and city level. Model (2) includes also varying slopes at the country level of the predictor Wave 2. Model (3) includes also country-level covariates to control for Covid-related variables such as number of cases and deaths (standardized). Model (4) includes interaction effect between Wave 2 and Age (to test Hypothesis 8) | | | | | | | | | | | | |
|  | **(1)** | | | **(2)** | | | **(3)** | | | **(4)** | | |
| *Predictors* | *Estimates* | *CI* | *p* | *Estimates* | *CI* | *p* | *Estimates* | *CI* | *p* | *Estimates* | *CI* | *p* |
| Constant | 0.154 | 0.141 – 0.167 | **<0.001** | 0.153 | 0.139 – 0.167 | **<0.001** | 0.153 | 0.140 – 0.165 | **<0.001** | 0.158 | 0.144 – 0.172 | **<0.001** |
| Wave 2 | -0.000 | -0.004 – 0.003 | 0.859 | -0.000 | -0.007 – 0.007 | 0.933 | -0.000 | -0.004 – 0.003 | 0.825 | -0.007 | -0.017 – 0.002 | 0.128 |
| Age | -0.001 | -0.001 – -0.000 | **<0.001** | -0.001 | -0.001 – -0.000 | **<0.001** | -0.001 | -0.001 – -0.000 | **<0.001** | -0.001 | -0.001 – -0.001 | **<0.001** |
| Gender | -0.057 | -0.061 – -0.054 | **<0.001** | -0.057 | -0.061 – -0.054 | **<0.001** | -0.058 | -0.061 – -0.054 | **<0.001** | -0.057 | -0.061 – -0.054 | **<0.001** |
| Student | 0.002 | -0.003 – 0.007 | 0.458 | 0.002 | -0.003 – 0.007 | 0.479 | 0.002 | -0.003 – 0.007 | 0.484 | 0.002 | -0.003 – 0.007 | 0.436 |
| Covid Cases (sd) |  |  |  |  |  |  | -0.007 | -0.020 – 0.006 | 0.297 |  |  |  |
| Covid Deaths (sd) |  |  |  |  |  |  | 0.002 | -0.011 – 0.015 | 0.788 |  |  |  |
| Wave2*Age |  |  |  |  |  |  |  |  |  | 0.000 | -0.000 – 0.001 | 0.118 |
| **Random Effects** | | | | | | | | | | | | |
| σ^2^ | 0.02 | | | 0.02 | | | 0.02 | | | 0.02 | | |
| τ_00_ | 0.00 _City_code_ | | | 0.00 _City_code_ | | | 0.00 _City_code_ | | | 0.00 _City_code_ | | |
|  | 0.00 _Country_ISO_Code_ | | | 0.00 _Country_ISO_Code_ | | | 0.00 _Country_ISO_Code_ | | | 0.00 _Country_ISO_Code_ | | |
| τ_11_ |  | | | 0.00 _Country_ISO_Code.Wave2_ | | |  | | |  | | |
| ρ_01_ |  | | | -0.37 _Country_ISO_Code_ | | |  | | |  | | |
| ICC | 0.05 | | | 0.05 | | | 0.04 | | | 0.05 | | |
| N | 43 _Country_ISO_Code_ | | | 43 _Country_ISO_Code_ | | | 42 _Country_ISO_Code_ | | | 43 _Country_ISO_Code_ | | |
|  | 55 _City_code_ | | | 55 _City_code_ | | | 54 _City_code_ | | | 55 _City_code_ | | |
| Observations | 29332 | | | 29332 | | | 28636 | | | 29332 | | |
| Marginal R^2^ / Conditional R^2^ | 0.036 / 0.084 | | | 0.036 / 0.088 | | | 0.039 / 0.079 | | | 0.036 / 0.084 | | |
| **Table S22.** Multilevel regression models of hygiene norms of brushing teeth. Model (1) considers varying intercepts at the country and city level. Model (2) includes also varying slopes at the country level of the predictor Wave 2. Model (3) includes also country-level covariates to control for Covid-related variables such as number of cases and deaths (standardized). Model (4) includes interaction effect between Wave 2 and Age (to test Hypothesis 8) | | | | | | | | | | | | |
|  | **(1)** | | | **(2)** | | | **(3)** | | | **(4)** | | |
| *Predictors* | *Estimates* | *CI* | *p* | *Estimates* | *CI* | *p* | *Estimates* | *CI* | *p* | *Estimates* | *CI* | *p* |
| Constant | 0.798 | 0.780 – 0.817 | **<0.001** | 0.801 | 0.782 – 0.820 | **<0.001** | 0.799 | 0.780 – 0.817 | **<0.001** | 0.801 | 0.782 – 0.819 | **<0.001** |
| Wave 2 | -0.003 | -0.006 – -0.001 | **0.015** | -0.004 | -0.011 – 0.003 | 0.249 | -0.003 | -0.006 – -0.000 | **0.024** | -0.008 | -0.015 – -0.000 | **0.039** |
| Age | 0.000 | 0.000 – 0.001 | **<0.001** | 0.000 | 0.000 – 0.001 | **<0.001** | 0.000 | 0.000 – 0.001 | **<0.001** | 0.000 | 0.000 – 0.001 | **0.002** |
| Gender | 0.017 | 0.014 – 0.019 | **<0.001** | 0.016 | 0.014 – 0.019 | **<0.001** | 0.017 | 0.014 – 0.020 | **<0.001** | 0.017 | 0.014 – 0.019 | **<0.001** |
| Student | 0.003 | -0.001 – 0.007 | 0.134 | 0.003 | -0.001 – 0.007 | 0.145 | 0.003 | -0.001 – 0.007 | 0.136 | 0.003 | -0.001 – 0.007 | 0.126 |
| Covid Cases (sd) |  |  |  |  |  |  | -0.012 | -0.037 – 0.013 | 0.348 |  |  |  |
| Covid Deaths (sd) |  |  |  |  |  |  | 0.023 | -0.002 – 0.047 | 0.072 |  |  |  |
| Wave2*Age |  |  |  |  |  |  |  |  |  | 0.000 | -0.000 – 0.000 | 0.204 |
| **Random Effects** | | | | | | | | | | | | |
| σ^2^ | 0.01 | | | 0.01 | | | 0.01 | | | 0.01 | | |
| τ_00_ | 0.00 _City_code_ | | | 0.00 _City_code_ | | | 0.00 _City_code_ | | | 0.00 _City_code_ | | |
|  | 0.00 _Country_ISO_Code_ | | | 0.00 _Country_ISO_Code_ | | | 0.00 _Country_ISO_Code_ | | | 0.00 _Country_ISO_Code_ | | |
| τ_11_ |  | | | 0.00 _Country_ISO_Code.Wave2_ | | |  | | |  | | |
| ρ_01_ |  | | | -0.31 _Country_ISO_Code_ | | |  | | |  | | |
| ICC | 0.22 | | | 0.23 | | | 0.22 | | | 0.22 | | |
| N | 43 _Country_ISO_Code_ | | | 43 _Country_ISO_Code_ | | | 42 _Country_ISO_Code_ | | | 43 _Country_ISO_Code_ | | |
|  | 55 _City_code_ | | | 55 _City_code_ | | | 54 _City_code_ | | | 55 _City_code_ | | |
| Observations | 29357 | | | 29357 | | | 28657 | | | 29357 | | |
| Marginal R^2^ / Conditional R^2^ | 0.005 / 0.224 | | | 0.005 / 0.234 | | | 0.021 / 0.232 | | | 0.005 / 0.224 | | |

**References**

1. Beugelsdijk, S. & Welzel, C. Dimensions and dynamics of national culture: Synthesizing Hofstede with Inglehart. *Journal of cross-cultural psychology* **49**, 1469–1505 (2018).

2. Inglehart, R. F., Ponarin, E. & Inglehart, R. C. Cultural Change, Slow and Fast: The Distinctive Trajectory of Norms Governing Gender Equality and Sexual Orientation. *Social Forces* **95**, 1313–1340 (2017).

3. Eriksson, K. *et al.* Perceptions of the appropriate response to norm violation in 57 societies. *Nature Communications* **12**, 1481 (2021).

4. Hofstede, G. Dimensionalizing cultures: The Hofstede model in context. *Online readings in psychology and culture* **2**, 2307–0919.1014 (2011).
